# Exploring Acylhydrazones’ Properties Against Neurodegenerative Diseases and Other Clinical Applications: A Review

**DOI:** 10.3390/ph19050679

**Published:** 2026-04-27

**Authors:** Julia Skroban, Marta Kruk-Słomka, Łukasz Popiołek

**Affiliations:** 1 Students’ Scientific Association at Chair and Department of Organic Chemistry, Faculty of Pharmacy, Medical University of Lublin, 4A Chodźki Street, 20-093 Lublin, Poland; julia.skr6@interia.pl; 2 Chair and Department of Pharmacology with Pharmacodynamics, Faculty of Pharmacy, Medical University of Lublin, 4A Chodźki Street, 20-093 Lublin, Poland; marta.kruk-slomka@umlub.edu.pl; 3 Chair and Department of Organic Chemistry, Faculty of Pharmacy, Medical University of Lublin, 4A Chodźki Street, 20-093 Lublin, Poland

**Keywords:** acylhydrazones, biological activity, enzyme inhibition, Alzheimer’s disease, cholinesterase inhibitors, MAO inhibitors

## Abstract

Neurodegenerative diseases are a serious problem for modern society, and their treatment remains an important issue discussed by the scientific community. One of the promising potential directions for modulating neurodegenerative processes is the use of acylhydrazones, a class of compounds that combine different bioactive fragments linked by an acylhydrazone moiety. So far, the biological properties of these compounds have been proven. They show antibacterial, antiviral, antifungal, antiparasitic, anticancer, anti-inflammatory and antioxidant activity. Many research papers focus on designing acylhydrazones that will find use in the treatment of neurodegenerative diseases by inhibiting the enzymatic activity of acetylcholinesterase (AChE), butyrylcholinesterase (BuChE), β-secretase 1 (BACE1) and monoamine oxidase (MAO), as well as inhibiting β-amyloid aggregation, exhibiting metal chelation and antioxidant properties. Recent studies have described the acylhydrazone-based dual (multi-target) inhibitors, which have demonstrated encouraging outcomes during *in vitro* evaluations. This review covers recent articles published in the years 2020–2025 and offers a comprehensive overview of the biological properties of the acylhydrazones and their multifunctional derivatives on neurodegenerative processes and/or neuroprotection, while emphasizing their universal nature, structural versatility and role as leading structures in the search for new drugs.

## 1. Introduction

Neurodegeneration is a chronic, progressive process that results in the gradual damage and death of nerve cells. This process is usually irreversible and leads to impaired brain or spinal cord function, manifesting as memory deficits and/or motor disturbances. Neurodegenerative diseases that develop as a result of these dysfunctions represent the most common heterogeneous group of chronic central nervous system disorders. These include, among others, Alzheimer’s disease (AD), Parkinson’s disease (PD), Huntington’s disease (HD), and amyotrophic lateral sclerosis (ALS) [1,2,3].

Despite differences in the clinical presentation of these diseases, the pathomechanism is shared, focusing on similar molecular mechanisms that lead to neuronal damage and death. The most common components of the pathomechanism of neurodegenerative diseases include excitotoxicity, energy deficits, oxidative stress, and inflammatory and immune responses [1,2,3].

Excitotoxicity is a pathological process associated with an excess of the excitatory amino acid glutamate (Glu), which, by stimulating its specific receptors, depolarizes the cell and allows the influx of calcium ions. Excess calcium is a fundamental factor determining the occurrence of neurotoxicity (activation of proteases generates damage to the cell membrane, activation of nitric oxide synthase and increased release of arachidonic acid led to increased free radical production and a cascade of adverse biochemical changes associated with oxidative stress). Another factor is energy deficit, meaning disturbances in the cell’s energy processes that lead to ion pump dysfunction, neuronal depolarization, increased calcium accumulation, and elevated free radical production. In turn, an excess of free radicals generates oxidative stress, which leads to damage at the level of nucleic acids, membrane lipids, and proteins. Another important mechanism in neurodegenerative diseases is their close association with inflammatory and immune processes. Chronic inflammation of nerve cells (neuroinflammation), particularly of microglia and astrocytes, leads to the release of pro-inflammatory cytokines and inflammatory mediators, promoting neuronal damage [1,2,3].

The most common neurodegenerative diseases include AD and PD. AD is the most common progressive neurodegenerative disorder, primarily affecting the loss of cholinergic neurons located in the forebrain, prefrontal cortex, hippocampus, and amygdala. Degeneration of cholinergic neurons leads to impaired cholinergic transmission due to a decrease in the concentration of acetylcholine (ACh), a neurotransmitter crucial for cognitive processes. Accelerated death of cholinergic neurons is caused by the production of two pathological protein deposits, which accumulate both within neurons and between them. The first pathological structure is the intracellularly deposited protein called β-amyloid (Aβ). Amyloid itself is a fragment of the amyloid precursor protein (APP), present in all brain cells, especially in high amounts during fetal life. The APP can be cleaved in different ways. Under physiological conditions, it is cut by α-secretase between the 16th and 17th amino acids of amyloid, preventing its release and the formation of harmful amyloid deposits. Under pathological conditions, cleavage is carried out by β-site amyloid precursor protein cleaving enzyme 1 (BACE1), a key enzyme in the pathogenesis of AD, and γ-secretase. The consequence of this is the formation of a 40–43 amino acid fragment that forms toxic Aβ deposits in the brain. Large Aβ particles form amyloid plaques, also called senile plaques, which deposit outside neurons and in the walls of capillaries, causing their damage and neuronal death. The second characteristic pathological structure in AD is the presence of hyperphosphorylated tau protein inside cells. Excess accumulation of free tau protein in the form of spirally twisted fibers causes microtubules to malfunction, and tau deposits prevent intracellular transport [4,5].

The decrease in ACh concentration in AD is a key therapeutic target in the pharmacotherapy of this disease. In the body, ACh is broken down by cholinesterase enzymes: acetylcholinesterase (AChE) and butyrylcholinesterase (BChE). AChE is primarily located in cholinergic synapses in the central nervous system, postganglionic synapses of the peripheral nervous system, and at the neuromuscular junction. BChE, on the other hand, is present in glial cells, especially in reactive microglia, but mainly outside synapses. Both enzymes are present in senile plaques, and inhibition of these enzymes results in increased ACh concentration in the synaptic cleft, thereby improving cognitive function in patients suffering from AD [6].

Parkinson’s disease (PD) is the second most common neurodegenerative disorder, second only to AD. In PD, dopaminergic neurons forming the nigrostriatal pathway degenerate, leading to a deficiency of dopamine (DA), a neurotransmitter essential for motor functions. The causes of dopaminergic neuron degeneration are diverse. The disease is believed to have a multifactorial basis, depending on both environmental causes and genetic predispositions. As a result of genetic mutations—mutations of α-synuclein in PD—so-called Lewy bodies are formed, which contain misfolded α-synuclein protein. α-Synuclein, by binding to neuronal membranes, leads to their damage, disturbances in cellular function, and cell death due to uncontrolled calcium influx, mitochondrial membrane depolarization, and impaired intracellular transport. DA deficiency leads to dysfunction of other neurotransmitter systems, particularly the cholinergic system. The resulting imbalance between DA and ACh causes the typical motor symptoms of PD (tremor, rigidity, bradykinesia) [7,8].

One of the therapeutic targets in PD is DA deficiency. In the cytosol of neurons, DA is degraded by two enzymes: monoamine oxidase (MAO) and catechol-*O*-methyltransferase (COMT). MAO exists in two isoforms, MAO-A and MAO-B, which differ in substrate specificity, tissue distribution, and substrate affinity. In humans, MAO-A is the dominant form in catecholaminergic neurons; however, with age, MAO-B activity and expression in the brain significantly increase (particularly in the striatum), promoting DA catabolism and contributing to Parkinsonian changes. MAO-B inhibitors increase DA levels in the brain by slowing its degradation in the synaptic cleft and enhancing DA availability [9].

The above-described mutually interconnected pathogenic processes form the basis for identifying key therapeutic targets. Enzymes such as AChE, MAO, and BACE1 are important targets for pharmacological intervention. Therefore, the design of multifunctional compounds, including acylhydrazones aimed at modulating/inhibiting multiple pathways simultaneously, represents a promising strategy for the treatment of neurodegenerative diseases.

Acylhydrazones are characterized by high plasticity and structural variability, which allows the synthesis of their derivatives with different biological properties. In organic chemistry, they are used as precursors for the synthesis of many heterocyclic compounds. As multifunctional ligands, acylhydrazones constitute a pharmacophore skeleton with proven therapeutic efficacy and potential for the development of new bioactive agents [10,11].

Although acylhydrazones have been known for many years and possess a vast range of biological activities (such as antibacterial, antiviral, antifungal, antiparasitic, and anticancer activity), there were no references about the potential use of acylhydrazones for the treatment of neurodegenerative diseases until the initial research on 8-hydroxyquinoline and isoniazid derivatives between 2013 and 2015 [12,13].

Most of the current research focuses on designing acylhydrazone that will be used in the treatment of neurodegenerative diseases by inhibiting the activity of acetylcholinesterase (AChE), butyrylcholinesterase (BuChE), β-secretase 1 (BACE1), monoamine oxidase (MAO), and β-amyloid aggregation, and exhibiting metal chelation and antioxidant properties. Recently, there have been reports of dual inhibitor (multi-target) acylhydrazones with promising results in preclinical studies [10,14].

This article aims to present recent publications describing the biological impact of acylhydrazones and their multifunctional derivatives on central nervous system functions and their potential use as new bioactive agents in the treatment of diseases associated with neurodegenerative processes.

## 2. Results and Discussion

Systematic research of leading scientific databases, including PubMed, ScienceDirect, Web of Science, Scopus and Google Scholar, was conducted using combined keywords such as: “acylhydrazones”, “central nervous system”, “neurodegenerative disease”, “neuroprotection”. Data analysis was performed based on predefined inclusion criteria (clinical trials, meta-analyses, full-text original and review articles published between 2020 and 2025).

### 2.1. Acylhydrazones in Medical Use

**Figure 1:**
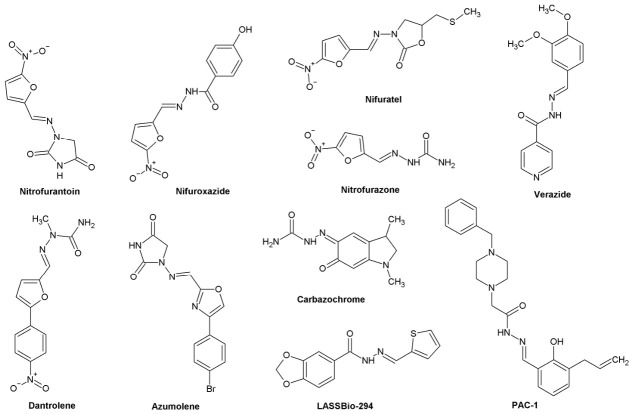
Chemical structure of acylhydrazones with documented biological properties used in the treatment or under clinical and pre-clinical investigations.

Compounds based on the acylhydrazone structure that are regulatory approved in therapies as current pharmacological standards (Figure 1):

○ **Nitrofurantoin**: Nitrofuran derivative used in the treatment and prevention of uncomplicated urinary tract infections,
○ **Nifuroxazide**: Nitrofuran derivative with local antibacterial activity, used in acute bacterial diarrhea,
○ **Nitrofurazone**: Antibacterial agent, mainly used topically on the skin (e.g., to treat burns or infected wounds),
○ **Nifuratel**: Nitrofuran derivative with antibacterial, antifungal and antiprotozoal properties, used in gynecology,
○ **Verazide**: Isoniazid derivative with antitubercular activity,
○ **Dantrolene**: Hydantoin derivative, ryanodine receptor antagonist in skeletal muscles, used in malignant hyperthermia,
○ **Azumolene**: Analog of dantrolene with better water solubility, similar indications as dantrolene,
○ **Carbazochrome**: Adrenochrome derivative, hemostatic (anticoagulant) agent [10,11,15,16,17,18].

Prospective substances under clinical and pre-clinical investigations (Figure 1):

○ **PAC-1** (**Procaspase-activating compound-1**): Activates PC-3 cancer cells and induces apoptosis, used in oncology (malignancies) [19],
○ **LASSBio-294** (**2′-thienylidene)-3,4-methylenedioxybenzoylhydrazine**): Calcium channel modulator and phosphodiesterase (PDE), used in heart failure and hypertension [20].

In the structure of acylhydrazones, we can distinguish the fragment –C(=O)–NH–N=CH–, which combines the chemical functions of the carbonyl, amide, hydrazine and imine groups, and give the molecules their special chemical properties. It contains both electrophilic centers (carbon atom in the imine group) and nucleophilic centers (nitrogen atom of the imine group with a free electron pair) as well as an acidic nitrogen atom (–NH–). Thanks to the above, acylhydrazone molecules may act as electron donors and acceptors, freely binding to receptors [21,22,23].

### 2.2. Isomeric Forms of Acylhydrazones

Acylhydrazones exist in four isomeric forms: geometric (*E*/*Z*) and conformational (*syn*/*anti*). Due to the presence of an imine group, acylhydrazones exhibit geometric isomerism, which directly affects the shape of the molecules and changes the way they form complexes with proteins. They occur as a mixture of *E* and *Z* isomers, with the *E* variant usually dominating. The presence of the *E* isomer ensures the stability of the *Z* variant. However, the existence of conformational isomers (*synperiplanar* and *antiperiplanar*) that form around the N-N bond allows them to form hydrogen bonds and chelate complexes with metal ions [24,25,26].

### 2.3. Synthesis Process of Acylhydrazones

**Figure 2:**
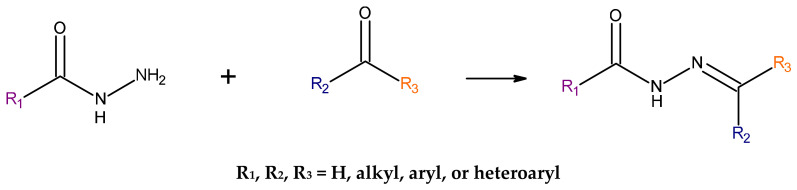
Standard procedure for synthesis of compounds from the acylhydrazone group.

The standard preparation method for acylhydrazones involves the condensation reaction of hydrazides of carboxylic acids with either ketones or aldehydes. This procedure is generally conducted in alcoholic solution under reflux conditions, facilitated by acidic, metal-based and organic (amino acids, ionic liquids) catalysts. Due to the crystalline nature of the resulting derivatives, they can be easily refined through re-crystallization (Figure 2) [11].

### 2.4. The Bioactivity of Acylhydrazones

#### 2.4.1. Inhibition of Cholinesterase Activity

One of the main strategies for the treatment of Alzheimer’s disease (AD) is the cholinergic strategy, which associates the appearance of clinical symptoms and their gradual progression with abnormal enzymatic activity of cholinesterases: acetylcholinesterase (AChE) and butyrylcholinesterase (BuChE) in the central nervous system (CNS). Cholinesterase inhibitors (IAChE) are the main group of drugs used in the symptomatic treatment of AD, but the clinical efficacy of drugs such as donepezil, rivastigmine and galantamine depends on the degree of neurodegeneration [27]. The search for new inhibitors with a broader spectrum of action therefore remains a valid direction for further research.

**Figure 3:**
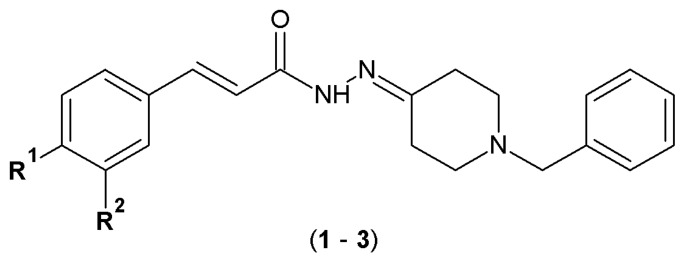
Acylhydrazones with potential application for the treatment of neurodegenerative diseases (**1**–**3**).

Ortiz et al. designed and synthesized a series of cinnamoyl-*N*-acylhydrazone-donepezil hybrids (**1**–**3**) as multifunctional ligands for the treatment of neurodegenerative diseases. The synthesis of molecular hybrids was based on combining a 1-benzyl-4-piperidine fragment (characteristic for donepezil) with a cinnamoyl subunit derived from curcumin (natural polyphenol with antioxidant, anti-inflammatory and neuroprotective properties). The acylhydrazone fragment acted as a linker with crucial role in the construction of these molecules (Figure 3) [28].

The colorimetric Ellman’s method was utilized to identify the inhibitory profile of the compounds against acetylcholinesterase (AChE) and butyrylcholinesterase (BuChE) enzymes. The ability of the compounds to scavenge free radicals was evaluated by mixing them with a DPPH radical solution (in ethanol) and measuring the absorbance. The lower the absorbance value, the greater the compound antioxidant property. The prevention of reactive oxygen species (ROS) of the tested derivatives was examined using 2′,7′-dichlorodihydrofluorescein diacetate (DCFH-DA) as a fluorescent probe/indicator. Results of the study demonstrated that compounds **1** and **3** (Table 1) exhibited moderate activity against AChE (IC_50_ of 13.04 and 9.1 µM, respectively, at the concentration of 30 µM). In addition, compounds **1** and **2** exhibited significant activity in the DPPH assay (80–90% ROS inhibition) and showed potential to protect neurons from oxidative stress induced by *tert*-butyl hydroperoxide (*t*-BuOOH) and 6-hydroxydopamine hydrochloride (6-OHDA). Compound **2** additionally reduced the formation of reactive oxygen species, increased intracellular glutathione levels (cells’ oxidative stress protection), decreased the neurotoxicity and neuroinflammation induced by OAβ1–42 (soluble oligomers with neurotoxic effects) and neurotoxin 6-hydroxydopamine (6-OHDA) (Table 1). Molecular docking results indicated that the binding modes of **1** and **3** were similar to that of donepezil: π-stacking interaction between the phenyl ring with the Trp86 indole ring, cation-π interaction of the piperidine group with the Tyr337 side chain, hydrogen bond between the carbonyl oxygen of the acylhydrazone group with the Tyr121 side chain, and hydrophobic interactions between the 1,2-dimethoxybenzene and the Peripheral Anionic Site (PAS) region and hydrophobic interactions between the methoxy at the *meta*-position with the Trp286 and Tyr72 side chains of PAS of AChE. In conclusion, the cinnamoyl-*N*-acylhydrazone-donepezil hybrids (**1**–**3**) represent promising candidates for the development of multifaceted neuroprotective agents with improved therapeutic efficacy [28].

**Figure 4:**
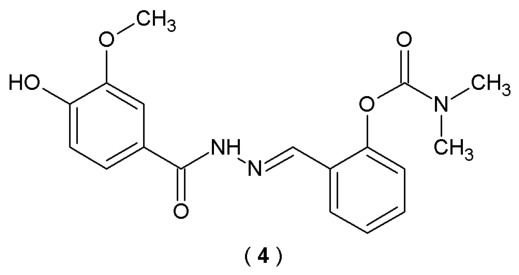
Arylcarbamate-*N*-acylhydrazone **4** with potential to inhibit cholinesterase enzymes.

Physostigmine was the first representative of the carbamate group clinically employed as an AChE inhibitor in the treatment of Alzheimer’s disease. Neostigmine and pyridostigmine are used in the management of myasthenia gravis and the treatment of glaucoma (neostigmine). Rivastigmine is the most important carbamate derivative available for the treatment of AD. On the other hand, the acylhydrazone scaffold is regarded as a privileged structure, capable of functioning as a pharmacophoric or auxophoric subunit across various classes of pharmaceutical compounds. In the search for novel bioactive agents, Yamazaki et al. synthesized and evaluated a series of arylcarbamate-*N*-acylhydrazone derivatives for their potential to inhibit cholinesterase enzymes. Their inhibitory activities against AChE and BuChE enzymes were tested using the modified colorimetric Ellman method. Concentrations of 10 and 100 µM were employed to evaluate the inhibitory activity of the compounds against both enzymes (Figure 4) [29].

Newly synthesized arylcarbamate-*N*-acylhydrazone derivatives exhibited remarkable butyrylcholinesterase inhibitory activity, with IC_50_ values ranging from 0.07 to 29.25 µM. The most potent compound among the series was acylhydrazone **4**, with IC_50_ values >100 µM for AChE and 0.07 µM for BuChE (fifty times higher than donepezil) (Table 2). Furthermore, kinetics analysis revealed that **4** acts as a non-competitive inhibitor of butyrylcholinesterase. Compound **4** possesses a carbamate group in the aromatic ring and a 3-methoxy-4-hydroxyphenyl scaffold linked with an *N*-acylhydrazone moiety. Based on the outcomes, the position of the carbamate group in the aromatic ring is related to the inhibitory activity of the studied compound. Molecular docking results indicated that compound **4** possesses the ability to form a strong hydrogen bond interaction with Tyr128, π-π stacking interaction with Trp82 and CH⋯O interactions with His438, Gly121 and Glu197. Therefore, compound **4** stands as the most promising leading structure for the development of effective AD treatment [29].

**Figure 5:**
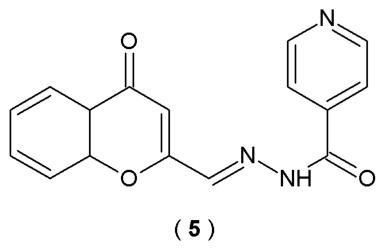
Chemical structure of isoniazide-based acylhydrazone **5** as a potential multi-target therapeutic agent for the treatment of AD.

Santos et al. investigated acylhydrazones derived from isoniazide as potential multi-target therapeutic agents for AD, a complex neurodegenerative disorder. Researchers designed and synthesized fifteen compounds. Scientists evaluated their ability to inhibit the enzymes myeloperoxidase (MPO) and AChE, relevant targets in AD pathology, and also tested their antioxidant properties (Figure 5). Antioxidant capacity of acylhydrazones was assessed at a concentration of 100 µM employing the DPPH stable free radical assay. To characterize the inhibitory profile of acylhydrazone compounds, their impact on the chlorinating activity of MPO (sourced from rat bone) was evaluated *via* the chlorotaurine-based assay. Inhibitory profile against AChE of the newly synthesized derivatives was identified by the modified colorimetric method [30].

Among all the synthesized substances, compound **5** demonstrated the most promising multi-target profile, showing significant inhibition properties. In the entire series, only compound **5** reduced more than 50% of AChE activity (54.2 ± 1.7% at 100 µM). Kinetic analysis confirmed that this compound acts as a non-competitive mixed inhibitor, which typically binds to an allosteric site in addition to the catalytic site (Table 3). Compound **5** major interactions within the AChE binding areas: the 3-chromonyl nucleus participates in π-π stacking with residues Tyr341, Phe297, and Trp286. Additionally, the stabilizing network is enhanced by hydrogen bonds formed between Ser293 and Phe295 and two specific acceptors: the ether oxygen of the 3-chromonyl scaffold and the carbonyl oxygen of the acylhydrazone group [30].

**Figure 6:**
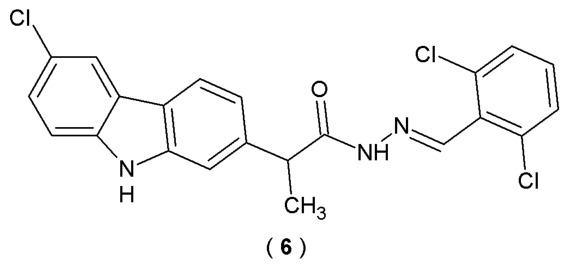
Chemical structure of acylhydrazone **6**.

Avram et al. utilized a microwave-assisted synthesis method to obtain the acylhydrazone derivatives, specifically (*EZ*)-*N*′-benzylidene-(2*RS*)-2-(6-chloro-9*H*-carbazol-2-yl)propanehydrazide (**6**). Synthesized compounds were structural hybrids of the carprofen molecule and belong to the family of Schiff bases due to the presence of the azomethine group (C=N) as a core pharmacophore (Figure 6). Compared to ordinary Schiff bases, these molecules are reported to be more stable, attributed to intramolecular hydrogen bond formation. Scientists assessed the pharmacokinetic potential of the *de novo* compounds by computing their permeability across the blood–brain barrier. The primary objective of this research was to assess the pharmacodynamic characteristics of newly derived acylhydrazones. To estimate potential macromolecular targets the Swiss Target Prediction and HitPick databases were used [31].

Compound **6** was identified as a potential agent in the treatment of neurodegenerative disorders and demonstrated blood–brain barrier permeability and inhibitory activity of CYP1A2, CYP2C19, and CYP2C9 (Table 4). Comparing the obtained compound with those already in use with the help of bioinformatics tools and chemical databases, scientists predicted the therapeutic utility of derivative **6** (potentially used in mental and behavioral disorders, mood disorders, and extrapyramidal and movement disorders) [31].

Patel et al. attempt to explore novel carbazole-based acylhydrazones derivatives’ abilities to inhibit cholinesterase and simultaneously demonstrate antioxidant properties. Carbazole derivatives are known to possess diverse activities relevant to AD, including cholinesterase inhibitory effects, Aβ aggregation abolishment and reactive oxygen species (ROS) scavenging properties. By combining the carbazole scaffold and the acylhydrazone moiety, the researchers created molecules with multiple beneficial properties. Using Ellman’s method, the study assessed the in vitro anticholinesterase potential of the new compounds. To ensure comparative accuracy, both donepezil and tacrine were utilized as benchmark controls [32].

**Figure 7:**
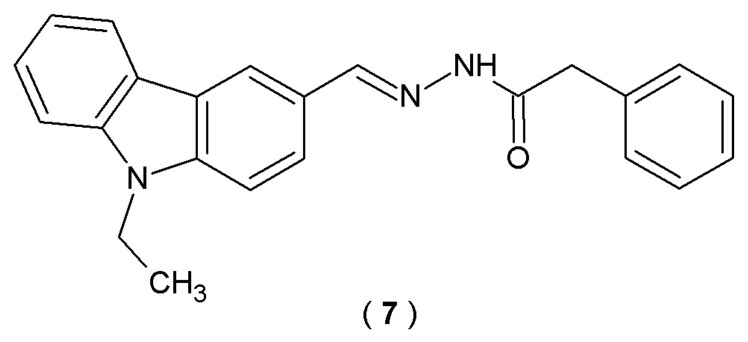
Acylhydrazone (**7**) with inhibitory effects on AChE and BuChE and antioxidant properties.

The research identifies (*E*)-*N*’-((9-ethyl-9*H*-carbazol-3-yl)methylene)-2-phenylacetohydrazide (**7**), which demonstrated good inhibitory activity (AChE and BuChE) and moderate radical scavenging properties confirmed by the experimental studies (Figure 7) [32].

Compound **7** demonstrated potent inhibitory activity for AChE (IC_50_ = 1.00 µM) and moderate inhibitory activity for BuChE (IC_50_ = 2.04 µM) in comparison with reference donepezil and tacrine. Compound **7** exhibited moderate radical scavenging abilities (Table 5). Taken as a whole, these results offer a promising starting point for designing versatile ligands. The ultimate goal is to produce therapeutic candidates that not only target AD symptoms but also hinder the overall progression of the AD [32].

**Figure 8:**
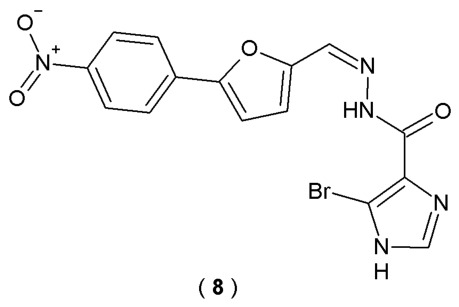
Acylhydrazone **8** with inhibitory effect on SOICR and AChE.

Disruption of calcium homeostasis and a reduction in acetylcholine concentration are recognized as two key mechanisms driving the progression of Alzheimer’s disease. Yang et al. searched for a compound that could concurrently suppress calcium oscillations (specifically, store overload-induced Ca^2+^ release—SOICR) and inhibit AChE activity, representing a promising and innovative therapeutic approach with renewed potential for effective AD management. The series of acylhydrazones incorporating aromatic substituents was synthesized and assessed in vitro for their inhibitory effects on SOICR and AChE (Figure 8). To monitor fluctuations in cytoplasmic Ca^2+^ levels, a calcium imaging assay was performed on RyR1R614C mutant cells. Simultaneously, the inhibitory effect on AChE was assessed *via* the Ellman method. In these experiments, dantrolene and donepezil served as positive controls, while dimethylsulfoxide (DMSO) was utilized as the negative control [33].

Among all of the synthesized acylhydrazones, compound **8** demonstrated high inhibitory potency against SOICR (73.6% inhibition at 10 μM) and AChE (29.1% inhibition at 5 μM) (Table 6) [33].

**Figure 9:**
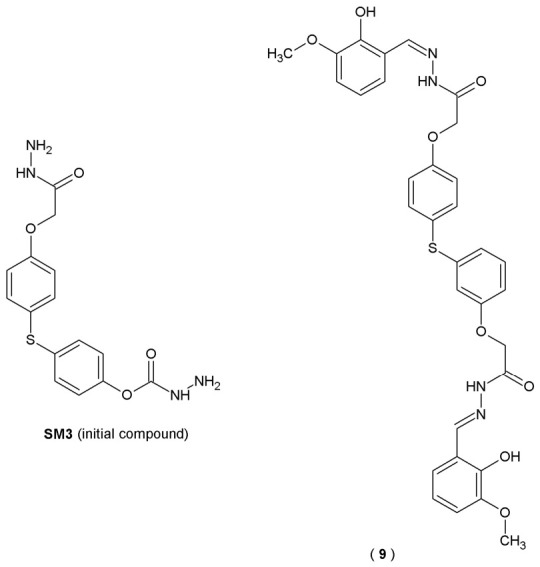
Acylhydrazone **9** and **SM3** with dual inhibitory properties on AChE and BuChE.

Both sulfide groups and acylhydrazones are important pharmacophores from a chemical point of view. Sulfide group-containing compounds possess a strong ability to inhibit multiple enzymatic processes. On the other hand, acylhydrazones (hydrazide-hydrazones), due to their structural stability and chemical reactivity, are widely used as intermediates in organic synthesis and possess a broad spectrum of biological activity. Reports indicate that both sulfide and hydrazide-hydrazone groups exhibit significant anticholinesterase activity. Therefore, a combination of a bisphenol sulfide and a hydrazide-hydrazone in a single molecule may further enhance their pharmacological properties. Research involving enzyme inhibition indicates that AChE and BChE work together to maintain cholinomimetic balance. Specifically, the observation that BChE levels increase as AChE activity declines points toward a compensatory mechanism for cholinergic degradation. This is supported by clinical data suggesting that inhibitors targeting both enzymes provide superior therapeutic outcomes compared to those selective for AChE alone. Based on this assumption, in their search for new cholinesterase inhibitors, scientists Ibrahim et al. synthesized a series of twenty-five 4,4′-thiodiphenol bis(acylhydrazones) based on the bisphenol sulfide skeleton (Figure 9). Their activity against acetyl- and butyrylcholinesterase was tested using a structure-based molecular docking approach [34].

Among all the examined derivatives, compound **SM3** (initial compound) and **9** exhibited dual inhibitor properties, effectively inhibiting both AChE and BuChE (values of AChE IC_50_ = 23.1 µM and BuChE IC_50_ = 21.8 μM for **SM3**, values of AChE IC_50_ = 27.8 µM and BuChE IC_50_ = 19.0 μM for compound **9**, respectively). For comparison, the IC_50_ value for galantamine, used as a reference drug, was 29.5 μM for AChE and 27.8 μM for BuChE (Table 7). Molecular docking analysis showed that **SM3** and **9** can form strong hydrogen bonds with the active sites of both enzymes. The results suggest that bis(acylhydrazones) may constitute potential alternative with therapeutic potential in the treatment of AD, comparable to the cholinesterase inhibitors currently in clinical use [34].

**Figure 10:**
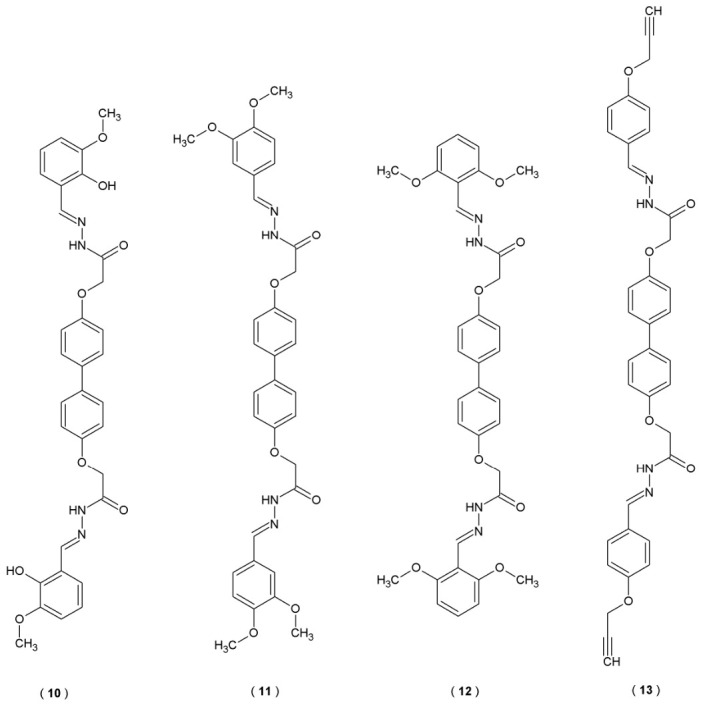
Bis(acylhydrazones) **10**–**13** with the ability to inhibit AChE and BuChE.

Further studies of Ibrahim et al. on the structure of bis(acylhydrazones) resulted in creating a new series of twenty-seven compounds, obtained by combining 4,4′-bisphenol and Schiff’s bases (Figure 10). Evaluation of these compounds’ anticholinesterase properties revealed significant inhibitory potential, which actually exceeded the performance of the standard drug, galantamine. These activities were measured through *in vitro* assays targeting both AChE and BChE enzymes [35].

Compounds **11** and **12** demonstrated strong inhibitory properties against AChE (IC_50_ = 26.3 and 28.4 μM, respectively). In terms of BuChE affinity, compounds **10** and **13** were confirmed as the most potent inhibitors (IC_50_ = 22.0 and 31.3 μM, respectively). Both derivatives were described as dual inhibitors and demonstrated stronger efficacy compared to the reference drug galantamine. Molecular docking confirmed the experimental observations, indicating stable binding within the active sites of the enzymes (Table 8). Additional structural modifications may enhance the potency of these compounds, potentially resulting in stronger inhibitory action against AChE and BuChE [35].

**Figure 11:**
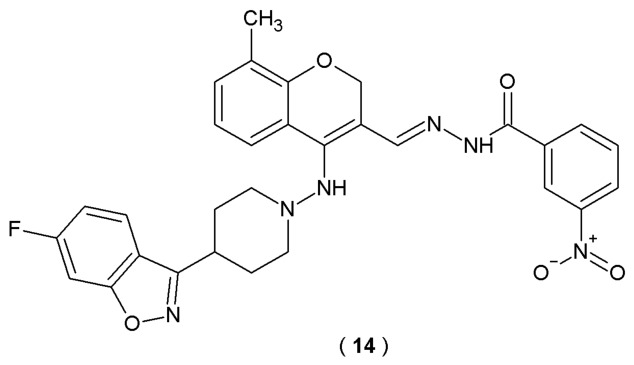
Chemical structure of acylhydrazone (**14**) as a potential acetylcholinesterase inhibitor.

The study held by Govada et al. successfully reported the synthesis and *in silico* evaluation of a new class of benzisoxazole-chromene acylhydrazone analogs as potential acetylcholinesterase inhibitors. Derivatives were obtained by the condensation of different benzohydrazides with benzisoxazole-chromenes (Figure 11). Molecular docking studies were performed on the newly synthesized molecules using the AChE enzyme. The structural data for the protein (PDB ID: 1H23) was sourced from the RCSB Protein Data Bank, while YASARA was utilized as the primary docking software. The docking procedure was conducted using the AutoDock Vina protocol within the YASARA environment. During protein preparation, extraneous molecules (including water and β-D-glucopyranose) were removed to ensure a clean binding site. To achieve a more refined clustering of the protein-ligand complexes, each compound underwent 100 Genetic Algorithm titration runs [36].

Among thirteen derivatives evaluated through molecular docking, nearly all exhibited favorable interactions and successfully bound within the active site of the 1H23 protein (AChE). According to the molecular docking results, derivative **14** exhibited strong hydrogen bond-forming ability (interactions with amino acid residues). Compound **14** was confirmed to be selective towards AChE, as proved by its binding affinity/efficiency (−13.39 Kcal/mol). Validation of the structure–activity relationships (SAR) of the novel BCA analogs highlights the importance of the combined scaffold, specific substituent patterns, and electronic properties for successful binding with active sites of acetylcholinesterase [36].

**Figure 12:**
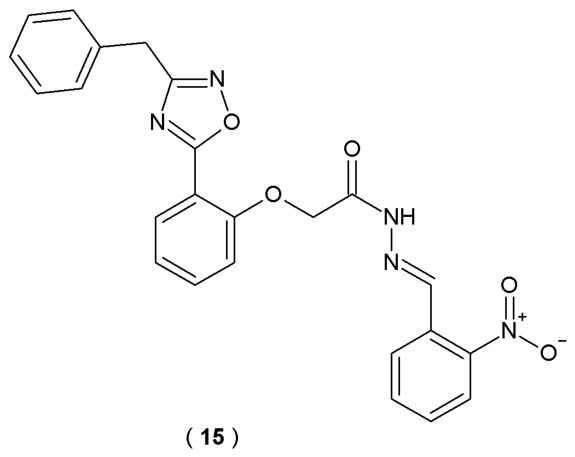
Chemical structure of new 1,2,4-oxadiazole derivative (**15**) with acylhydrazone moiety with inhibitory activity towards cholinesterases and antioxidant properties.

Ayoup et al. developed a series of novel 1,2,4-oxadiazole-based derivatives as potential multifunctional anti-AD agents (Figure 12). Specialized configuration design of the 1,2,4-oxadiazole scaffold is recognized in the scientific community for developing innovative chemical structures due to its distinct bioisosteric characteristics. To assess how the newly synthesized oxadiazole derivatives perform against cholinesterase enzymes, their IC_50_ concentrations were measured. The anti-AD drugs donepezil and rivastigmine served as benchmarks for this comparison of inhibitory strength. The antioxidant capacity of the synthesized oxadiazole derivatives was measured using a DPPH radical scavenging assay. Quercetin served as the reference drug, a well-established antioxidant standard. The study evaluated the inhibitory potential of all synthesized 1,2,4-oxadiazole derivatives against both MAO-B and MAO-A enzymes. To provide a clear benchmark for these activities, biperiden and methylene blue (methylthioninium chloride) were utilized as reference standards [37].

Among synthesized substances, compound **15** was identified as a promising drug candidate due to its high activity and good physicochemical properties (Table 9). Compound **15** showed the highest *in vitro* inhibitory activity, achieving an exceptionally low IC_50_ value of 0.00098 µM. The potency of these compounds was significantly higher than that of donepezil, with an efficacy level 1.55 times greater than the reference drug. Introduction of the benzyl moiety at position 3 of the oxadiazole ring resulted in increased activity towards AChE. The MAO-A inhibitory effects of oxadiazole **15** were notable, showing a 1.4-fold increase in potency compared to methylene blue. These findings position compound **15** as a highly promising candidate for subsequent Alzheimer’s disease research and therapeutic development [37].

Bartolić et al. designed and synthesized the novel hydrazone and acylhydrazone derivatives of vitamin B_6_ and pyridine-4-carbaldehyde, targeting hallmarks of neurodegeneration. Scientists based the selection of the core structural scaffold on the fact that the acylhydrazone moiety shares structural similarity with the amidine moiety found in potent β-secretase (BACE1) inhibitors like Verbecestat and Atabecestat. The amidine structure in these two agents governs their binding affinity *via* multiple hydrogen bonds with the catalytic aspartates in the BACE1 active site. The novel acylhydrazone derivatives of pyridoxal compounds were evaluated for their inhibitory potential against human cholinesterases (AChE and BuChE), BACE1 and amyloid self-aggregation inhibition abilities [38].

**Figure 13:**
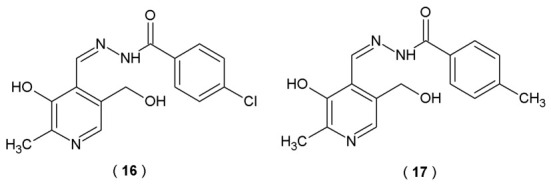
Chemical structure of acylhydrazones **16** and **17** with inhibitory activity towards AChE and BuChE and antioxidant properties.

The outcomes revealed that a series of *N*-acylhydrazone derivatives reversibly inhibited human AChE with inhibition constants (Ki ranging from 89 to 199 µM) in biological evaluation studies. The incorporation of substituents at the benzohydrazide position yielded a marginal enhancement in the compound’s inhibitory efficacy. The most potent AChE inhibitor in the group was compound **16** (4-chlorohydrazone derivative), which was twice as potent as the unsubstituted precursor. The BuChE inhibition potency of *N*-acylhydrazone derivatives increased with the addition of halogen substituents (**16**) compared to the unsubstituted analogs. Compound **17**, with an electron-donating methyl group, was the most potent BuChE inhibitor (Figure 13, Table 10) [38].

1. **Inhibition potency towards BACE1.** BACE1 inhibition profiles of the synthesized compounds were quantified by calculating the percentage of enzymatic suppression. Results were derived from differential fluorescence signals recorded in the presence of inhibitors (FAB, BACE1, substrate and tested hydrazones with concentrations of 10µM and 50µM, respectively) to those of the non-inhibited control reactions (FAB, BACE1 and substrate). Inhibition activity of BACE1 of both compounds was within the range of 10–69% [38].

2. **Ability to inhibit amyloid-β (Aβ42) aggregation.** The inhibitory potential of acylhydrazone derivatives was evaluated *via* the Thioflavin T fluorometric assay. By monitoring the shift in fluorescence emission, scientists track the extent to which compounds suppress the spontaneous fibrillization process of Aβ42 peptides. Compound **16** demonstrated the highest amyloid self-aggregation inhibition among all the derivatives, decreasing amyloid aggregation by approximately 36%. Compound **17** reduced Aβ42 self-aggregation by 23% [38].

3. **Metabolic stability.** Only compound **16** was selected for *in vitro* studies in human liver microsomes due to its high inhibitory potency against AChE, BChE, and BACE1 with moderate metabolic stability [38].

The sulfonate ester linked with fluorinated acylhydrazone is a hybrid structure synthesized by integrating commercially available 4-fluorobenzoic hydrazide and aryl sulfonates. These compounds contain four significant pharmacophore moieties in their structures, fluorine atom, methoxy group, sulfonate fragment, and acylhydrazone fragment, which can be found in various medical products used in AD and glaucoma. Specifically, the research centered on their efficacy against human carbonic anhydrase I and II (hCA I, hCA II) and cholinesterases (AChE, BuChE). Akis et al. designed and synthesized a series of hybrid molecules derived from aryl sulfonate ester intermediates and performed extensive *in vitro* biological assays to measure their potency [39].

**Figure 14:**
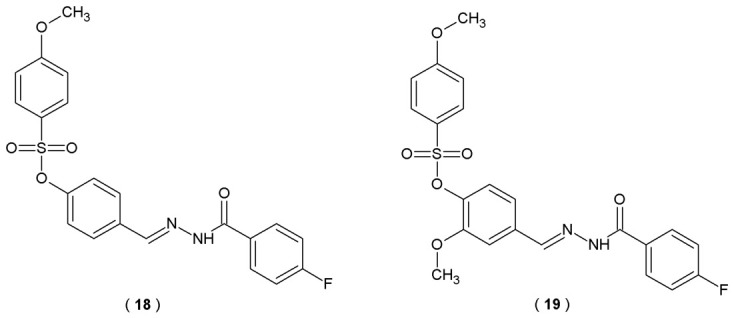
Chemical structure of acylhydrazones (**18**, **19**) with efficacy against human carbonic anhydrase I and II (hCA I, hCA II) and cholinesterases (AChE, BuChE).

Sulfonate ester-linked fluorinated acylhydrazone derivatives with linear structure **18** and **19** were identified as the most potent inhibitor candidates (Figure 14, Table 11). Compound **19** was the most active inhibitor against hCA I (IC_50_ = 30.4 µM), nine times more potent than the reference Acetazolamide and hCA II (IC_50_ = 23.2 µM). Superior activity against hCA I and hCA II of this substance is attributed to the electron-donating and activity-enhancing methoxy group located in the fifth position of the phenyl ring in the spacer moiety. Compound **18** demonstrated the strongest inhibitory effect against AChE (IC_50_ = 12.1 µM) and was nearly eleven times more active than the reference substance neostigmine and almost five times more than rivastigmine. In conclusion, acylhydrazones **18** and **19** stand as viable candidates in the search for anti-AD treatments, warranting more extensive investigation and optimization in future studies [39].

**Figure 15:**
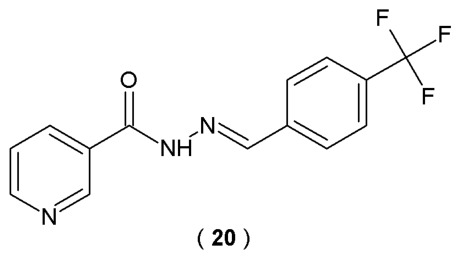
Chemical structure of isoniazid-based acylhydrazone **20**.

Frias et al. searched for compounds that exhibit biological activity against AD biomarkers. They presented a synthesis and detailed characteristics of new isoniazid-based acylhydrazones, studying their effect on AChE activity, β-secretase (BACE-1) and the β-amyloid (Aβ) aggregation process. In this study, an inhibitory potential of compounds against acetylcholinesterase (AChE) and β-secretase (BACE-1) was evaluated using Ellman’s colorimetric method and a FRET-based assay. Additionally, Thioflavin T fluorescence emission was employed to monitor Aβ fibril formation, while the DPPH radical scavenging method was utilized to determine the antioxidant capacity of the compounds. The synthesis of acylhydrazones was carried out by condensing isoniazid with appropriately substituted aromatic aldehydes (Figure 15) [40].

In this study, most of the synthesized acylhydrazones exhibited significant activity against AChE. The highest efficacy was observed for compound **20** (IC_50_ = 2.98 µM). Additionally, compound **20** demonstrated strong affinity for β-secretase (BACE-1) compared to the reference quercetin (IC_50_ = 63.9 µM). Compound **20** possesses moderate ability to counteract amyloid deposition (IC_50_ = 17.1 µM) but the highest antioxidant activity (88%), which confirms its biological potential (Table 12). Given that Alzheimer’s disease is a multifactorial condition, this compound was presented as a viable prototype for the design of more advanced therapeutic candidates [40].

In later studies, Ibrahim et al. designed and synthesized a series of twenty-nine 4,4′-sulfinyldiphenol-linked acylhydrazone derivatives, which were tested *in vitro* for their activity against acetyl- and butyrylcholinesterase. The inhibitory activities against AChE and BuChE were quantified spectrophotometrically *via* Ellman’s assay. In this procedure, acetylthiocholine iodide and butyrylthiocholine iodide served as enzymatic substrates. The presence of the 5-thio-2-nitrobenzoate anion was confirmed by the emergence of a yellow color, which occurs when thiocholines react with the DTNB reagent. In the synthesis of bis(acylhydrazones) scaffolds, 4,4-dithiophenol served as the initial compound [41].

**Figure 16:**
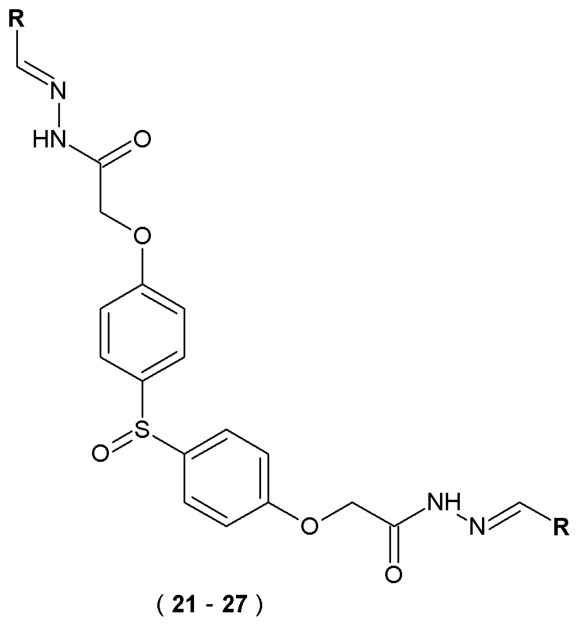
Chemical structures of acylhydrazones (**21**–**27**).

The highest activity against AChE was observed for compounds **22**, **24**, **25** and **26** with the presence of hydroxyl and methoxyl groups in positions 2, 3, 4 and 5 (Figure 16). Compound **25** in particular showed selective activity against AChE. A similar activity profile was observed for BuChE. Among the compounds substituted at position four, only compound **21** achieved an IC_50_ value (53.9 μM) comparable to galantamine (57.1 μM), while the other analogs were significantly weaker (IC_50_ = 77.8–376.6 μM). These results indicate that substitutions at position four (*para*) of the aromatic ring are generally disadvantageous for BuChE inhibition. The most active BuChE inhibitors were compounds **21**, **23**, **25**, **26** and **27**. Compounds **21** and **23** acted as selective BuChE inhibitors, while substances **25**, **26** and **27** exhibited dual inhibitory activity against AChE and BuChE. Furthermore, the derivatives showed a stronger inhibitory effect on enzymes than galantamine, which can be explained by their larger molecular size, allowing for more effective interaction with the active site of enzymes. For reference, a smaller compound of a galantamine molecule has a limited ability to bind similarly (Table 13). These results indicate that the molecules possess significant drug-like potential, suggesting they could serve as viable candidates following further optimization [41].

**Figure 17:**
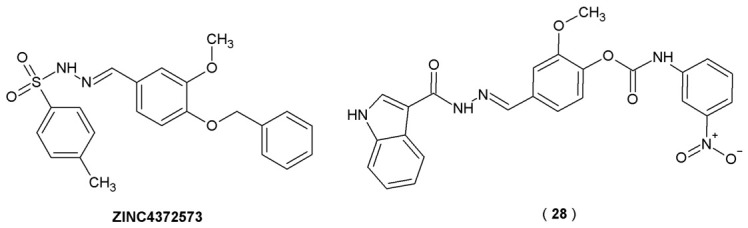
Chemical structures of compounds **ZINC4372573** and acylhydrazone **28**.

By utilizing computer-aided drug design techniques, molecular docking and molecular dynamics simulations, researchers identified **ZINC4372573** as a lead compound with high theoretical activity and binding affinity (Figure 17). To identify novel lead candidates through computer-aided drug design, the crystal structure of AChE (PDB ID: 4EY6) was obtained from the RCSB Protein Data Bank for use as the receptor model. The co-crystallized ligand, galantamine, served as the positive control to validate the docking parameters. Following rigorous preparation of the target and library, molecular docking was executed *via* SYBYL-X 2.122. This workflow enabled the virtual screening of a library comprising roughly 500,000 compounds from the ZINC database. Based on this lead and using vanillin as a core scaffold, Li et al. synthesized a series of vanillin derivatives as potential dual-target inhibitors for Alzheimer’s disease. The presence of the vanillin scaffold allows these novel compounds to bind with the active sites of acetylcholinesterase through facilitating interactions with the Peripheral Anionic Site (PAS) of AChE. Specifically, the vanillin core is responsible for the compound’s ability to form π−π interactions with the amino acid residue Tyr341 in the PAS domain. This interaction is essential for preserving the compound’s physiological activity and stabilizing the ligand–enzyme complex. Beyond its structural utility, vanillin was selected because it is a naturally occurring organic compound with antioxidant and neuroprotection-enhancing abilities [42].

The anticholinesterase potential of compound **28** was determined through the Ellman colorimetric assay, targeting both AChE and BuChE with galantamine as the reference standard. The *in vitro* assay results showed that compound **28** exhibits the most potent inhibitory activity against both AChE and BuChE among all tested compounds (with IC_50_ values of 0.18 μM and 7.61 μM, respectively), surpassing the positive control drug galantamine (Table 14, Figure 17). Structural and kinetic studies confirm that **28** binds effectively to the target enzymes, suggesting a mixed-type inhibition mechanism for AChE. Furthermore, **28** demonstrated notable antioxidant activity in ABTS radical scavenging assay with 2,2′-azino-bis(3-ethylbenzothiazoline-6-sulfonic)acid measured by calibration curve, with an activity (0.40) that is comparable to natural antioxidants like polyphenols and carotenoids (0.02–0.7) and slightly lower than that of ascorbic acid (0.99). This suggests potential benefits of the molecule in regulating oxidative stress processes. In light of these results, compound **28** emerges as a potent dual-target inhibitor of AChE and BuChE, representing a significant lead for the design of innovative Alzheimer’s disease therapeutics [42].

#### 2.4.2. Inhibition of Monoamine Oxidase Activity

**Figure 18:**
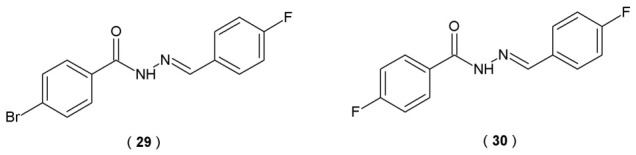
Chemical structures of compounds **29** and **30**.

Monoamine oxidase (MAO), particularly the MAO-B isoform, is involved in the pathophysiology of many diseases. The activity of the MAO enzyme increases with age and also in the course of neurodegenerative diseases (for example, AD or PD), making it a potential therapeutic target. Jayan et al. identify two acylhydrazones, **29** and **30**, with outstanding inhibitory potency against MAO-B (Figure 18). The enzymatic activities of acylhydrazones on MAO-A and MAO-B were assessed through continuous spectrophotometric assay (25 °C for 30 min). Absorbance changes were monitored at 316 nm and 250 nm, respectively. These assays utilized kynuramine (0.06 mM) for MAO-A and benzylamine (0.30 mM) for MAO-B within a 0.5 mL reaction volume, buffered by 50 mM sodium phosphate (pH 7.2). Evaluating the capacity of CNS-active agents to traverse the blood–brain barrier is essential for their therapeutic efficacy. In this study, the Parallel Artificial Membrane Permeability Assay (PAMPA) was employed to estimate the passive transcellular diffusion of the tested compounds. This cell-free methodology provides a robust predictive model for assessing how effectively a drug candidate might penetrate the BBB. Molecular dynamics (MD) simulations were conducted in a physiological environment to simulate the behavior of the compound **29** within the MAO-B binding pocket [43].

Pharmacokinetics study results confirmed that both molecules acted as competitive, reversible MAO-B inhibitors, modulating enzyme activity. Respective inhibition constant values (Ki) were determined to be 0.097 μM for **29** and 0.10 μM for **30** (Table 15). Furthermore, compounds **29** and **30** effectively penetrated the blood–brain barrier in the Parallel Artificial Membrane Permeability Assay (PAMPA) assay. Both demonstrated significant CNS permeability, yielding *P_e_* values higher than 4.0 × 10^−6^ cm/s, which indicates favorable permeability and bioavailability profiles for brain-targeted delivery. *In silico* docking simulations indicated that acylhydrazone **29** interacts with MAO-B protein residues through hydrophobic forces, stabilizing the enzyme–ligand complex. Structural analysis revealed that Tyr398 maintained a π−π stacking interaction with a 90% occupancy rate. Furthermore, Gln206 created stable hydrogen bonds with the NH moiety of the hydrazone group within the complex. These findings highlighted acylhydrazones **29** and **30** as promising candidates for the management of neurological disorders [43].

**Figure 19:**
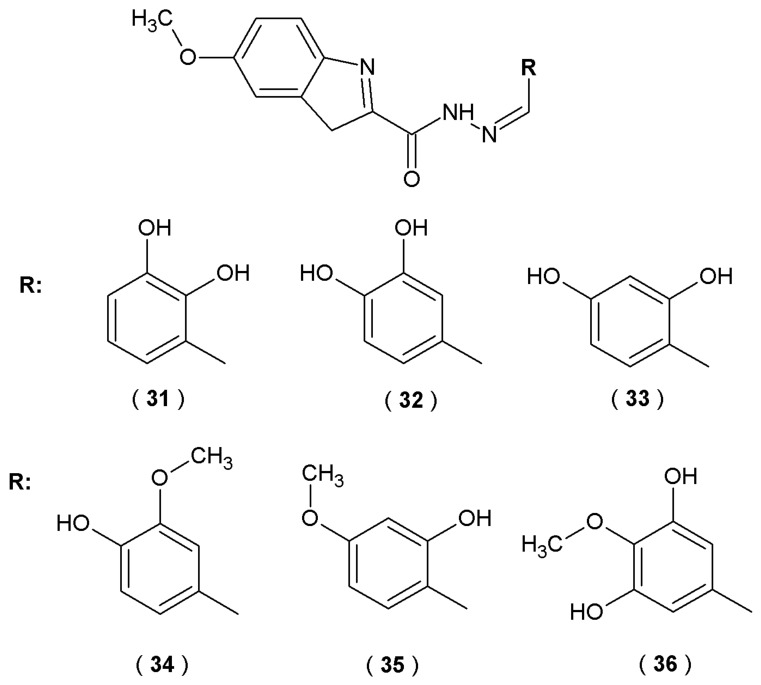
Indole-based acylhydrazones chemical structures (**31**–**36**).

The research of Anastassova et al. focused on the evaluation of new indole derivatives with acylhydrazone moiety as potential agents for neurodegenerative disorders (Figure 19) [44].

The inhibitory effects on recombinant human MAO-B (hMAO-B) were evaluated using a fluorometric approach. In this essay, the enzymatic reaction generates hydrogen peroxide, which subsequently reacts with Amplex UltraRed reagent (10-acetyl-3,7-dihydroxy-phenoxazine) in a 1:1 stoichiometric ratio in the presence of peroxidase. This process yields resorufin, a red phosphorescent oxidation product, which was quantified spectrophotometrically by leveraging its high extinction coefficient. The compounds (**31**–**36**) showed significant inhibition of recombinant human MAO-B (hMAO-B) activity (35–40% at a concentration of 1 µM) compared to reference IPA (indole-3-propionic acid) (25%) but not as good as standard rasagiline or selegiline (50–55%). Molecular docking results revealed that these novel derivatives freely bind with the flat hydrophobic cavity of the MAO-B active site, similar to the reference substances such as melatonin and rasagiline. Especially, compounds **35** and **36** were found to be situated within the MAO-B active site, specifically localized between the Tyr398 and Tyr435 residues that constitute the aromatic cage. This spatial orientation is essential for aligning natural substrates with the FAD cofactor. Consequently, these acylhydrazones effectively mimic the optimal position of the scissile bond during enzymatic catalysis. The capacity of the target compounds to scavenge superoxide anion radicals was evaluated *via* a spectrophotometric xanthine oxidase assay. This method measured the compounds’ ability to inhibit the radical-induced reduction of nitroblue tetrazolium (NBT). The antioxidant potential was screened at concentrations of 1, 10, 50, and 100 µM. A significant 20% decrease in the absorbance ratio was noted for derivative **32** at 10 µM compared to the control group. A significant reduction in the superoxide-anion species accumulation during performer assays was observed, suggesting that this molecule possesses a promising inhibitory effect on reactive oxygen species (ROS) generation. To investigate the neuroprotective potential of the synthesized agents, their capacity to traverse the blood–brain barrier (BBB) was evaluated. Scientists utilized a static *in vitro* model featuring the bEnd3 microvascular endothelial cell line. This static permeation assay was essential to confirm that the compounds could transition from systemic circulation to the CNS, thereby fulfilling the requirement for intracranial therapeutic activity. Derivatives **34** and **35** showed higher relative potency by decreasing permeability at 10 µM, while compounds **31**, **32** and **33** required a higher concentration of 50 µM to achieve the same results. Based on the observed antioxidant effects alongside effective MAO-B suppression, acylhydrazones emerge as promising multifunctional agents for the management of Parkinson’s disease [44].

**Figure 20:**
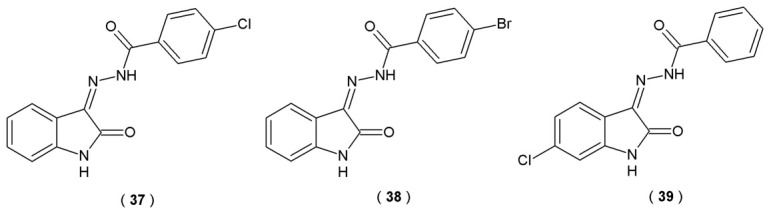
Acylhydrazones (**37**–**39**) with the ability to inhibit monoamine oxidase activity.

Kumar et al. described the synthesis and biological evaluation of sixteen isatin-tethered halogen-containing acylhydrazones as potential therapeutic agents for neurological disorders (Figure 20). The scientists focused on exploiting the C-3 position of the isatin structure, as isatin is known to bind close to the flavin adenine dinucleotide (FAD) cofactor in the MAO-B substrate cavity. They incorporated an acylhydrazone linker at the C-3 position as a structural core containing two distinct nitrogen atoms. To further enhance MAO efficacy, the C-3 position was substituted with a halogenated phenyl (hydrophobic) moiety. Enzymatic activities of novel acylhydrazones on MAO-A and MAO-B were quantified using 0.06 mM kynuramine and 0.3 mM benzylamine as substrates, respectively. Continuous spectrophotometric method (benzaldehyde UV detection) at 250 nm allowed for the assessment of compound potency, which was benchmarked against the standard inhibitors toloxatone and clorgyline [45].

The results of performed assays revealed acylhydrazones **37**, **38** and **39** as potent, selective, and reversible inhibitors of MAO-B. Compound **38** turned out to be the most potent inhibitor of MAO-B, with a value of 0.082 µM, followed by **39** (0.104 µM) and **37** (0.124 µM) (Table 16). All three acylhydrazones demonstrated high permeability across the blood–brain barrier in the PAMPA, indicating potential good CNS bioavailability. Their CNS permeability results in P_e_ > 4.00 × 10^−6^ cm/s, which is comparable to the reference drug selegiline. Compounds **37**–**39** were also evaluated *in vitro* to assess their protective role against LPS-induced inflammation. Using the human neuroblastoma SH-SY5Y line, the study aimed to quantify how these molecules modulate the cellular response to inflammatory stress. Researchers utilized MTT viability assays and enzyme-linked immunosorbent assays (ELISA) and measured changes in the levels of IL-6, TNF-α and NF-κB to determine the compound’s ability to modulate inflammatory signaling. Results indicated that the tested acylhydrazones (**37**–**39**) exhibited neuroprotective and anti-inflammatory effects during *in vitro* studies. Molecules significantly increased antioxidant levels and decreased pro-inflammatory cytokines (IL-6, TNF-α, NF-κB). In summary, the results suggest that the lead derivatives **37**–**39** represent promising therapeutic candidates for the management of various neurological conditions [45] (Table 16).

**Figure 21:**
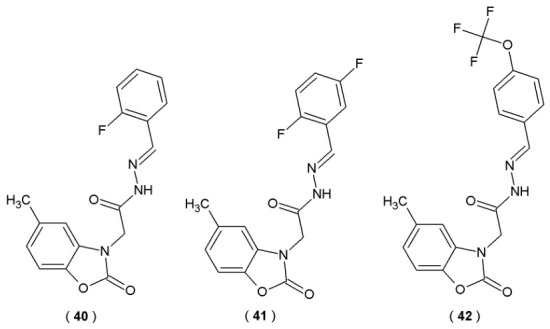
Chemical structure of acylhydrazones (**40**–**42**).

Taşci et al. focused on the development of the novel acylhydrazones with potential activity against monoamine oxidases: A (MAO-A) and B (MAO-B). Three acylhydrazones were obtained (**40**–**42**) (Figure 21). The activity of the novel compounds was evaluated against MAO-A and MAO-B enzymes at concentrations of 10^−3^ and 10^−4^ M. Inhibitory potency of these derivatives was quantified by comparing the inhibition results to reference drugs such as selegiline and moclobemide [46].

All tested compounds demonstrated significant activity against MAO-B, achieving inhibition levels of 69–77% at higher concentrations (10^−3^ M). In the case of MAO-A, the inhibitory effect was significantly weaker (<50%) (Table 17). Despite demonstrating favorable *in silico* binding orientations, experimental data indicated that compounds **40**–**42** lacked *in vitro* inhibitory potency. Molecular docking results revealed that electrostatic interactions play the most significant role in maintaining the structural stability of the molecules. Nevertheless, the acylhydrazone scaffold remains a promising pharmacophore for MAO targeting, suggesting that further structural optimization is justified [46].

**Figure 22:**
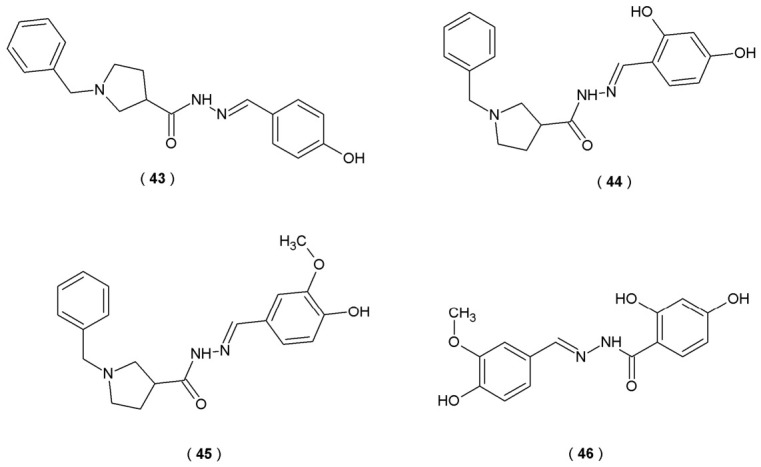
Acylhydrazones **43**–**46** with potential activity towards MAO-A and MAO-B.

The main purpose of the work of Kondeva-Burdina et al. was to evaluate two series of hybrid compounds with the ability to inhibit MAO-A and MAO-B, critical in neurochemical balance. The compounds were synthesized as hybrid molecules of the main therapeutic agents: melatonin and donepezil. This hybrid design allows the molecules to simultaneously target multiple pathological pathways, aiming to restore neurotransmitter balance and counteract neurodegeneration (Figure 22). In this study scientists utilized a high-sensitivity method, an Amplex Red Hydroxyl Peroxidase (HRP) Assay Kit, to measure peroxidase activity or hydrogen peroxide concentrations in biological environments. HRP assay provides an accurate quantitative measurement of the enzymatic activity of newly developed acylhydrazones. This technique involves the Amplex Red reagent (10-acetyl-3,7-dihydroxyphenoxazine), which serves as a fluorogenic probe. When peroxidase is present, 10-acetyl-3,7-dihydroxyphenoxazine is oxidized into resorufin, a red-tinted product. This transformation occurs with a strict 1:1 stoichiometry, meaning the resulting red-fluorescent signal is directly proportional to the amount of hydrogen peroxide consumed. Researchers also evaluated how effectively acylhydrazones inhibited the CYP3A4 isoform of human recombinant cytochrome P450 using a standard inhibitor screening kit. In this model, resorufin acted as the substrate, and the well-characterized inhibitor ketoconazole was used as a reference to measure relative inhibitory activity [47].

The study outcomes identified derivative **44** as a selective MAO-B inhibitor (0.524 ± 0.20 µM). However, derivatives **43**, **45** and **46** exhibited potent activity against both MAO isoforms, classifying them as dual MAO-A and MAO-B inhibitors (Table 18). This dual action suggests their potential as versatile modulators of monoaminergic pathways. All five of the most active compounds (**43**–**46**) demonstrated protein–ligand interaction of the arene–arene type with Tyr435. Specific interactions observed in the case of selegiline, such as interaction with Ile199, were found in the enzyme-binding model of compound **45**. Nevertheless, this study revealed that tested compounds exhibited moderate CYP3A4 inhibition (potential drug–drug interactions). Specifically, derivatives **43**–**46** demonstrated notable inhibitory properties as the CYP3A4 isoform inhibitors. Experimental results indicated that acylhydrazones reduced enzymatic activity to roughly 70% of the control group’s capacity (an approximate 30% inhibitory effect), identifying them as active leads in the screening process. The identified compounds may serve as excellent lead structures in the development of next-generation neuroprotective drugs [47].

#### 2.4.3. Other Biological Properties

Santos et al. reported acylhydrazones derived from isoniazid as potential multi-target therapeutic agents for AD. Researchers synthesized fifteen compounds and evaluated their ability as inhibitors of the enzymes myeloperoxidase (MPO) and AChE as well as antioxidant properties. The antioxidant efficacy of the acylhydrazones was evaluated by the radical scavenging (DPPH) assay at 100 µM, a common preliminary test for antioxidant activity. Antioxidant activity is quantified by the decrease in DPPH absorbance, a change attributed to the hydrogen-donating capacity of the tested compounds. The ability of the acylhydrazones to inhibit MPO-mediated chlorination was assessed using a chlorotaurine-based assay. Myeloperoxidase derived from rat bone marrow served as the enzymatic source for the screening, with isoniazid employed as a positive control for benchmarking inhibitory efficacy. The research findings indicate that compound **5** has the most promising multi-target profile (Figure 5, Table 3). Compound **5** exhibited high antioxidant capacity (DPPH Assay), with 82.2 ± 1.7% scavenging at 100 µM and an IC_50_ value of 42.4 ± 1.9 µM. Moreover, the myeloperoxidase inhibitory activity of compound **5** reached a value of 80.1 ± 9.4% at 10 µM (IC_50_ = 5.3 ± 0.5 µM), comparable to isoniazid (IC_50_ = 3.9 ± 0.3 µM). According to molecular docking studies, the amino substituents located at the terminus of the indole-attached alkyl chain of the compound establish hydrogen bonds with Glu102 and increase the MPO activity [30].

**Figure 23:**
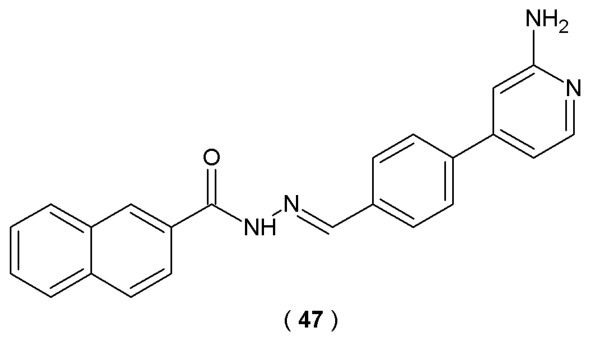
Chemical structure of amino-pyridinyl-*N*-acylhydrazone (**47**) with potential anti-inflammatory activity.

Cordeiro et al. performed a synthesis of new 2-amino-pyridinyl-*N*-acylhydrazones derivatives with anti-inflammatory activity, specifically compound **47** and its hydrochloride analog with improved solubility. Researchers developed these compounds as potential anti-inflammatory drugs by modifying LASSBio-1824 (p38α MAPK inhibitor). They utilized non-radioactive enzyme-linked immunosorbent assay (ELISA) to monitor p38α MAPK activity of the novel compounds. In this assay antibodies specifically detect phosphorylated mitogen-activated protein kinases. SB 203580 was selected as the reference inhibitor due to its established efficacy against the target enzyme. The specific anti-inflammatory mechanisms of action of compound **47** and its hydrochloride consist of modulating the p38 mitogen-activated protein kinase (MAPK) pathway and reducing acute inflammatory mediators (TNF-α, IL-1, IL-6) (Figure 23) [48].

The experimental outcomes revealed that **47** demonstrates weak inhibitory activity on p38 MAPK (IC_50_ = 40.6 µM). In contrast to compound **47**, modest improvement of inhibitory potency was observed for the hydrochloride analog (IC_50_ = 28.4 µM) (Table 19). Notably, **47** hydrochloride analog showed high potency in reducing IL-1β secretion (>90%) at higher doses. Both compounds significantly reduced *in vivo* nitric oxide (NO) levels in the SAP model. Subsequently, NO reduction was confirmed *in vitro*, where both compounds significantly reduced NO produced by the activated cell line RAW 264.7. Both compound **47** and its hydrochloride analog required further studies to discover their potential medical implementations [48].

**Figure 24:**
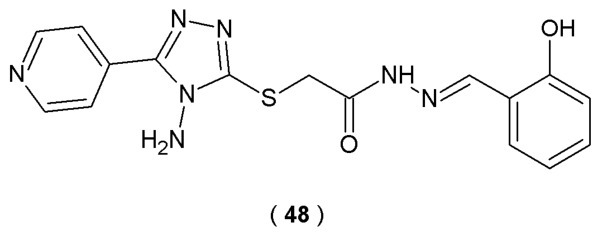
The 1,2,4-triazole derivative (**48**) with activity towards Neuro-2a cells.

Jiang et al. focused their work on the neurite outgrowth-promoting potential of a novel series of acylhydrazone compounds containing the 1,2,4-triazole fragment. In this study, Neuro-2a cells served as an *in vivo* model for neuronal cell differentiation. The neuronal differentiation effects of the acylhydrazone series were examined by treating Neuro-2a cells with 5 µM of each derivative for 48 h. By culturing cells in 0.5% FBS, scientists promoted an environment conducive to differentiation, allowing them to capture structural changes using phase contrast microscopy. Subsequent data analysis prioritized the differentiation frequency and the longest neurite extension per cell as indicators of successful neuronal development. The targeted acylhydrazones, synthesized by condensing isoniazide with an aldehyde or ketone, possess strong coordination ability and exhibit a wide spectrum of biological activities. The primary chemical novelty of molecules is the combination of the acylhydrazone moiety with the 1,2,4-triazole structure (Figure 24) [49].

Scientists discovered that compound **48** demonstrates superior neurite outgrowth-promoting activity and suggested that it operates through the PI3K-Akt and MEK-ERK signaling pathways. Results showed that only compound **48** increased the differentiation level of Neuro-2a cells at a concentration of 1–10 µM for 48 h. Derivative **48** exhibited stronger effects on neurite outgrowth promotion than positive control retinoic acid, including the differentiation rate (>40%) and the longest neurite length (>70%). Compound **48** (concentration of 5 µM) significantly increased the phosphorylation levels of extracellular signal-regulated kinases 1/2 (ERK1/2) and p38 in Neuro-2a cells in the first 30 min of treatment. The findings collectively demonstrated that compound **48** induces neuronal differentiation and neurite extension through the activation of MEK-ERK and PI3K-Akt signaling pathways. These results indicate that compound **48** promotes neural regeneration and has high potential in the treatment of neural injury and neurodegenerative diseases [49].

**Figure 25:**
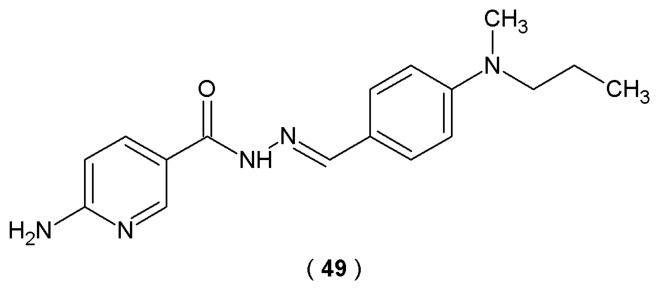
Chemical structure of pyridine derivative **49**.

ERRγ is a nuclear receptor, expressed in various structures of the central nervous system. It is responsible for regulating metabolic functions and the processes of differentiation of nervous cells. The work of Kim et al. aimed to design new agonists of the estrogen-dependent receptor with the potential ability to activate CREB (cAMP response element-binding protein). CREB is a cellular transcription factor that activates the expression of dopaminergic neuron markers: tyrosine hydroxylase (TH) and dopamine transporter (DAT). A series of thirty-six acylhydrazones based on the structure of the ERRγ agonists GSK4716 and DY131 was developed by introducing various dialkylamino groups in the *para* position of the aromatic ring to investigate their effect on biological activity, as previous ERRγ agonists had a single substituent in this position (Figure 25) [50].

Transcriptional activity against ERRγ was investigated based on the ability of the novel compounds to activate the ERRγ ligand-binding domain (luciferase assay). The most promising outcomes were observed for substance **49**, surpassing the reference agonist GSK4716. Data analysis of cells derived from neuroblastoma cancer cell lines (SH-SY5Y), treated with 5 μM of **49**, confirmed that the compound increased the levels of TH and DAT—markers of dopaminergic neurons (immunocytochemistry for TH *p* < 0.0001 and DAT *p* < 0.001 compared with the untreated group). In addition, compound **49** significantly increased the level of phosphorylated CREB (Ser133), correlated with the intensity of dopaminergic neuron differentiation processes (immunocytochemistry for p-CREB *p* < 0.001 compared with the untreated group). Molecular docking studies results revealed that the presence of the 2-aminopirydynyl group in the structure of compound **49** allows the formation of an additional hydrogen bond with the Glu247 residue of ERRγ binding domain. These outcomes demonstrated the ability of the novel ERRγ agonist to form stronger hydrophobic interactions with receptors than GSK4716, which explains their biological activity [50].

**Figure 26:**
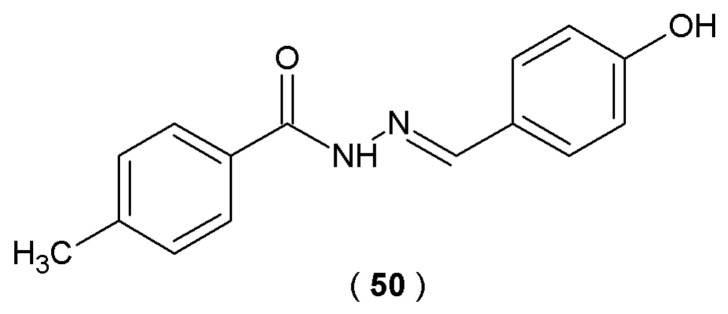
Chemical structure of 4-methylbenzohydrazide derivative (**50**) with binding affinity for bovine serum albumin.

The research of Zhang et al. explored the binding properties of aromatic 4-methylbenzohydrazide derivatives. The researchers synthesized compound **50** as a lead structure and investigated its potential interaction with bovine serum albumin (BSA). The target compound was prepared by condensing the intermediate 4-methylbenzohydrazide with 4-hydroxybenzaldehyde (Figure 26). Interaction between the BSA molecule and 4-methylbenzohydrazide derivative was verified using a dual-analytical approach involving UV-visible spectroscopy and electrochemical impedance spectroscopy (EIS). Molecular docking simulations of the novel acylhydrazones were conducted using the AutoDock 4 software suite [51].

Experimental results indicate that compound **50** exhibits moderate binding affinity with bovine serum albumin. The bovine serum albumin molecule has two main binding sites: subdomain IIA (Sudlow’s site I) and subdomain IIIA (Sudlow’s site II). Derivative **50** binds selectively to Sudlow’s site II of serum albumins. Molecular docking outcomes confirmed the role of electron interactions, hydrophobic interactions and hydrogen bonds in stabilizing the **50-BSA** complex (binding energy value of -7.19 kcal/mol^−1^). Compound **50** has one binding site on BSA, confirmed through the double logarithmic model. The presence of hydroxyl group on the phenyl ring of **50** enhances the molecule’s activity toward BSA [51].

Besides enzymatic inhibitory activity against MAO-B, indole-based acylhydrazones investigated by Anastassova et al. proved to have neuroprotective abilities tested in various cell models [35]. Derivatives **31**–**36** demonstrated high neuroprotection potency, confirmed by preserving synaptosomal viability (SV) and reducing glutathione (GSH) levels in 6-OHDA induced neurotoxicity test. The 2,3-dihydroxyphenyl- (**31**), 2-hydroxy-4-methoxyphenyl- (**35**), and syringaldehyde (**36**) derivatives provide an especially high level of SV (56%). Moreover, derivative **32** managed to decrease the amount of superoxide-anion radical generation by over 20% at 100 µM, tested spectrophotometrically by xanthineoxidase assay. In conclusion, synthesized compounds were considered promising candidates for the treatment of neurodegenerative disorders based on their combined effects of MAO-B inhibition and oxidative stress reduction (Figure 19) [44].

**Figure 27:**
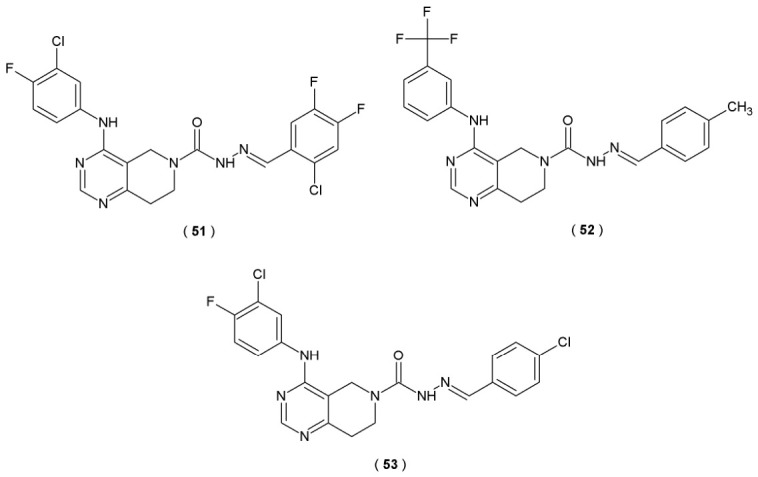
Chemical structure of *N*-acylhydrazones (**51**–**53**) with ATX inhibition activity.

Autotaxin (ATX) is identified as a glycoprotein enzyme that catalyzes the production of lysophosphatidic acid (LPA) from lysophosphatidylcholine (LPC). ATX inhibitors regulate the ATX-LPA signaling pathway associated with various disorders (e.g., chronic inflammation, fibrosis, cancer progression). The study held by Zhang et al. identifies acylhydrazones as linker moieties of novel dual ATX and EGFR inhibitors (Figure 27). The structures of compounds **51** and **52** were based on the EGFR inhibitor gefitinib, substituting the parental tetrahydropyrido[4,3-d]pyrimidine with a 4-aminoquinazoline scaffold and simultaneously incorporating a semicarbazone moiety (Figure 27). Researchers identify potential ATX antagonists through high-throughput screening (HTS) assay. This technique measures the conversion of lysophospholipase D to lysophosphatidylcholine, a reaction mediated by lysophosphatidic acid enzyme. Fluorescence-based assays represent the most widely adopted screening method with the synthetic probe FS-3 serving as a standard tool for detecting changes in autotaxin-mediated signaling and inhibition [52].

Compounds **51** and **52** exhibited substantial inhibition potency of both ATX (IC_50_ values of 38.4 nM and 29.1 nM, respectively) and epidermal growth factor inhibitor (EGFR), confirming their potential as double kinase inhibitors. Both compounds demonstrated anti-fibrotic and anti-proliferative abilities, substantiated by *in vitro* assays. To further augment the suppression activity of ATX, Zhang et al. incorporated the halogen substituents at the terminal position of the linker and phenyl chains. This process led to the creation of compound **53**. The study outcomes confirmed that compound **53** decreased the collagen levels by 74.4%, demonstrating anti-fibrotic potency and counteracting the TGF-β-mediated cardiac fibrosis. Furthermore, compound **53** prevented the accumulation of collagen in mice, resulting in remission of the CCL4-induced liver fibrosis (Table 20) [52].

**Figure 28:**
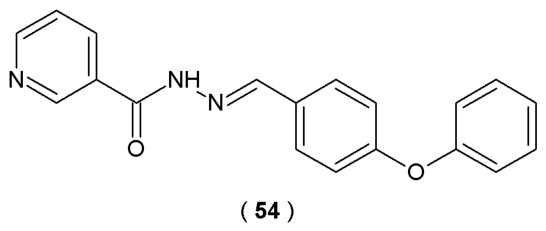
Nicotinic acid derivative (**54**) with inhibitory properties towards FAAH.

The primary objective of the research conducted by Gür Maz et al. was to explore the inhibitory potency of new acylhydrazones derived from nicotinic hydrazide against human Fatty Acid Amide Hydrolase (FAAH). FAAH is an enzyme implicated in regulating the endocannabinoid system and linked to conditions such as inflammation and neurological disorders (Figure 28), confirmed *via* spectroscopy method. FAAH activity was monitored using a fluorometric technique based on the hydrolysis of the substrate by FAAH. In this model, the resulting fluorescence signal correlates directly with the level of enzyme activity, which provides a robust and highly sensitive platform for screening potential inhibitors [53].

Compound **54**, the acylhydrazone analog with a 4-phenoxyphenyl group, was confirmed to be the most effective FAAH inhibitor among the series (Figure 28). The acylhydrazone led to suppression of the FAAH activity by 40.4% at 10 µM and 23.7% at a concentration of 1 µM in biological evaluation studies. Molecular docking revealed that compound **54** interacts with the active sites of the enzyme: forming two π-π stacking bonds with TRP531, one with PHE381 and one hydrogen bond with THR488 [53].

**Figure 29:**
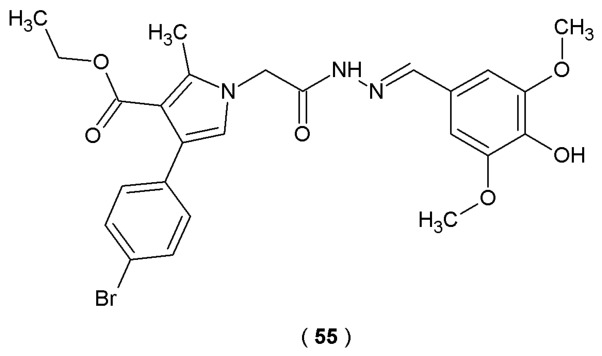
Pyrrolylhydrazide derivative (**55**) with antioxidant properties.

Tzankova et al. conducted *in vitro* evaluations of the antioxidant properties of novel *N*-pyrrolylhydrazide-hydrazones using DPPH and ABTS assays to confirm the theoretical findings. The experimental results highlighted compound **55** as the strongest antioxidant with the best results in the radical scavenging assay (Figure 29). This effect was primarily attributed to the introduction of a *para*-hydroxyl group in the phenyl fragment of the molecule. Moreover, the study examined cytotoxicity and protective effects of the compounds against H_2_O_2_-induced oxidative stress in human neuroblastoma SH-SY5Y cell lines [54].

Compound **55** demonstrated the highest DPPH scavenging activity (24%). At the same time, derivative **55** exhibited superior efficiency in a model of H_2_O_2_-induced oxidative stress in SH-SY5Y cells (30–60% cells protection) compared to the reference melatonin (20–50%). In conclusion, compound **55** was characterized as the lead compound of the series because it demonstrated superior radical scavenging abilities with antioxidant protective effects in both DPPH and H_2_O_2_-induced oxidative stress models with the lowest cellular toxicity. As such, acylhydrazone **55** provides a strategic starting point for a future series of molecules, with the ultimate goal of enhancing inhibitory potency through structural modification [54].

**Figure 30:**
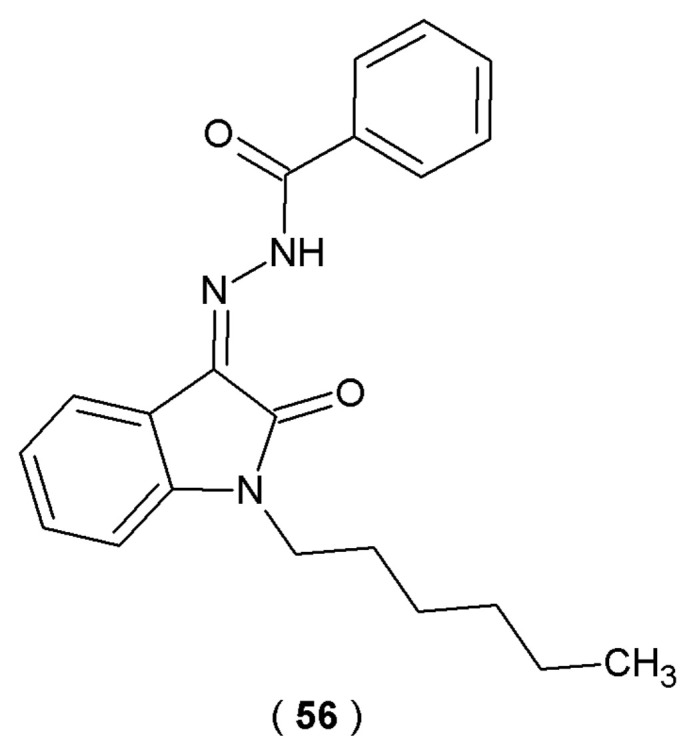
Acylhydrazone **56** with potential anti-inflammatory effects.

CB1R and CB2R, as members of the G protein-coupled receptor (GPCR) superfamily, are two primary subtypes of cannabinoid receptors. The scientific evidence confirmed that activation of CB2 receptors modulates immune responses by promoting the secretion of cytokines such as IL-6 and IL-10 in human leukocytes, while downregulating the expression of IL-17, IFN-γ, TNF-α, IL-6, and IL-12 in immune effector cells. This combined regulatory activity contributes to the receptor’s anti-inflammatory effects. In conclusion, CB2R-selective agonists have notable therapeutic potential in the management of neuroinflammatory conditions, cardiovascular diseases, and cancer. Zhang et al. introduced a series of *N*-alkyl isatin acylhydrazones with potential inhibitory effect on CB2R receptors (Figure 30). By measuring the capacity of a test compound **56** to competitively displace a standardized radioactive ligand from the CB2R binding site, researchers determined the inhibitory constant (Ki) and selectivity profile of the synthesized derivatives [55].

Radioligand competitive binding assays carried out by scientists revealed that compound **56** exhibited strong binding affinity toward CB2R (Kᵢ = 44.3 ± 10.2 nM) *via* the incorporation of *n*-hexane and phenyl groups into its molecular structure. Further research demonstrated EC_50_ values of 63.4 ± 1.3 nM for this compound. Biological evaluations indicated that compound **56** acts as a selective CB2R agonist and constitutes a prospective treatment of neuropathic pain (Table 21) [55].

Neural stem/progenitor cells (NSPCs) are present both during the embryonic phase and in the fully grown central nervous system. They play an important role in neurogenesis due to their self-renewal and proliferation abilities. In order to understand the molecular mechanisms of induced neurogenesis, the researchers used high-throughput transcriptome sequencing technology (Illumina RNA-seq). A compound known as **48**, an acylhydrazone with a 1,2,4-triazole moiety, was developed by Jiang et al. as part of a series of acylhydrazones containing a 1,2,4-triazole structure. Compound **48** was identified as a novel neurogenic agent with the potential to promote neural regeneration (Figure 24). This molecule significantly affects neurogenesis and induces the differentiation of neural stem cells into neurons and astrocytes. These outcomes were confirmed by observing higher percentages of cells positive for the neuronal marker (tubulin III) and the astrocyte marker (GFAP) [56].

When tested on Neuro-2a cells, compound **48** promoted neuronal differentiation and neurite outgrowth in a concentration-dependent manner. Notably, compound **48** exhibited stronger activities than the positive control, retinoic acid (RA), regarding both the differentiation rate and the longest neurite length. Transcriptome analysis revealed that the studied compound promotes the up-regulation of genes associated with neurogenesis while simultaneously inhibiting genes related to cell cycle progression. Presented results show that genes with increased expression (Gata3, Helt, Flil, Neurod2, Neurod6, Neurog2, Zfp488, Nhlh1, Myt1, Egr3, Irx3, Egr1, Olig1, Neurod1, Myrf, Olig2, Scrt1, Hey2, Foxp4, Sox1, Zfp977, Zfp618 and Sox3) were primarily associated with neurogenesis processes, while genes with reduced expression were responsible for cell cycle regulation (CDK1, CyclinA2, CyclinB1, Cyclin E1). Observations indicated that acylhydrazone **48** activity may be associated with the modulation of key regulatory genes (MAP2 and NeuN) [56].

**Figure 31:**
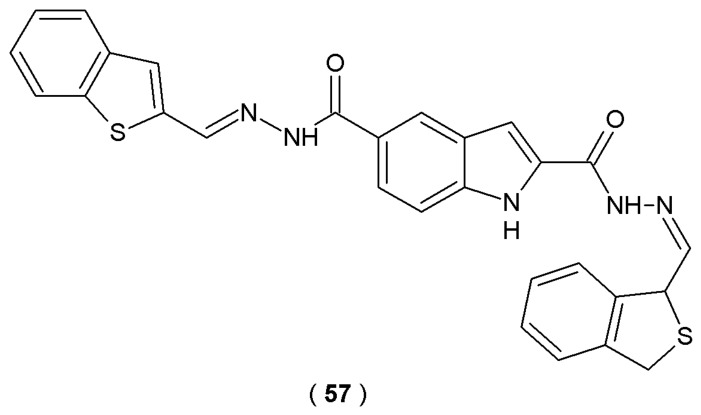
Chemical structure of acylhydrazone **57** with the ability to inhibit α-glucosidase activity.

Based on the comprehensive experimental and theoretical data presented by Jiang et al., compound **57** showed the highest affinity for α-glucosidase among all tested compounds, with the lowest Ki value of 4.56 μM (Figure 31). The study outcomes determined the mixed inhibition mechanism of indole-based bis(acylhydrazone) compounds on α-glucosidase (non-covalent forces stabilizing the 1:1 complex). Researchers used theoretical approaches to confirm that competitive binding to the active site of α-glucosidase (single binding site) is the dominant factor contributing to the potent antidiabetic activity of compound **57** (Table 22) [57].

**Figure 32:**
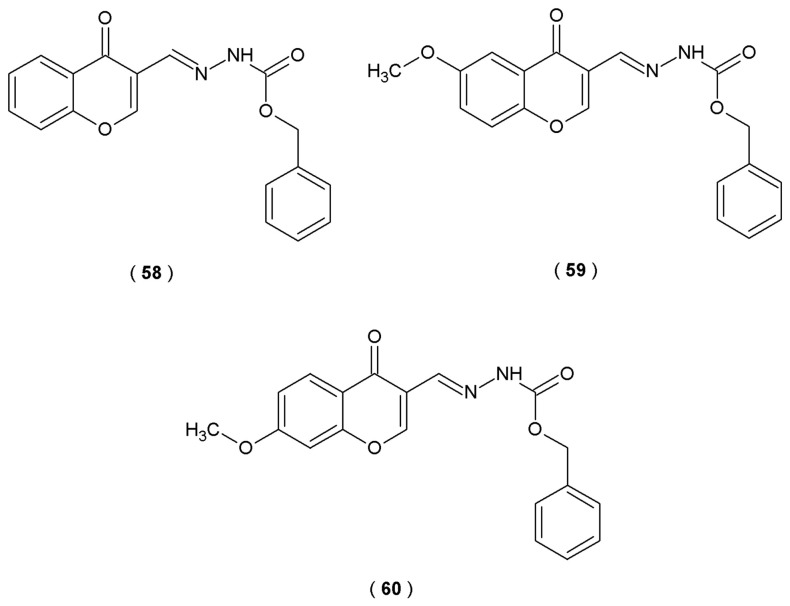
Chromone-based acylhydrazones **58**, **59** and **60** with COX inhibition properties.

Elzahhar et al. designed and synthesized a new series of chromone-based derivatives incorporating a carbamate acylhydrazone moiety in their molecular structure. The efficacy of these novel derivatives resides in their mechanisms of action, simultaneously suppressing the action of the arachidonate pathway targets, cyclooxygenase (COX-2), lipoxygenase (15-LOX), and microsomal prostaglandin E2 synthase 1 (mPGES), exerting anti-inflammatory effects. The naturally derived chromone main scaffold was selected as the central framework, substituted at positions 6–7 with a methoxy group. The *N*-acylhydrazone fragment was incorporated because it is considered a privileged structure, recognized as an important pharmacophore for COX inhibition and previously identified as an inhibitor of mPGES-1 (Figure 32). In this research, the COX-1/COX-2 inhibitory profile of novel acylhydrazones was determined by the ovine COX-1/human recombinant COX-2 assay kit. Experimental results were compared against established positive controls, including celecoxib (selective inhibitor), indomethacin and diclofenac (non-selective inhibitor) [58].

The new chromone-based compounds, specifically the benzylcarbazates (**58**–**60**), display similar anti-inflammatory potency to established drugs like celecoxib and diclofenac. Compound **58** demonstrated COX-2 inhibitory activity (IC_50_ = 0.049 µM), comparable to the selective COX-2 inhibitor celecoxib (IC_50_ = 0.045 µM). In addition, the 15-LOX inhibitory activity of compound **58** was higher than that of reference quercetin (IC_50_ = 1.72 ± 0.03 µM). Biological evaluation studies identify compound **60** as the most potent inhibitor of the mPGES-1 (IC_50_ = 2.80 µM). Furthermore, it proved to be more selective COX-2 inhibitors than both reference drugs, diclofenac and indomethacin. The primary results indicate that the benzylcarbazate analogs are potent, multi-target anti-inflammatory agents with superior efficacy and an acceptable safety profile (Table 23) [58].

**Figure 33:**
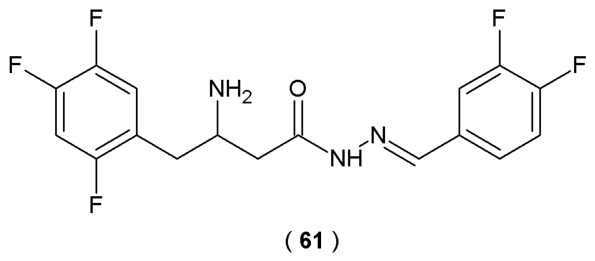
Chemical structure of acylhydrazone (**61**).

The study of Reina et al. aimed to design and evaluate new β-amino-*N*-acylhydrazones as dipeptidyl peptidase IV (DPP-4) inhibitors. These derivatives were the result of a hybridization process between the C-subunit of sitagliptin and LASSBio-1773. Utilizing both theoretical modeling and *in vitro* assays, the research investigated five crucial structural variables. The *R*-configuration of the amino group and the *E*-geometry of the imine double bond were critical for maximum inhibition of the DPP-4 enzyme. Compound **61** was successfully identified as a new lead with favorable physicochemical properties, high inhibitory potency, and proven anti-diabetic effects in an *in vivo* model (Figure 33). The hybrid structure of **61** was developed through the reaction of 2,4,5-trifluorophenylacetic acid with Meldrum’s acid in the presence of carbonyl diimidazole (CDI). The crucial transformation involved classical isosterism, replacing the sulfonyl (SO_2_) group with the carbonyl (C=O) group to yield the final *N*-acylhydrazone framework [59].

**Figure 34:**
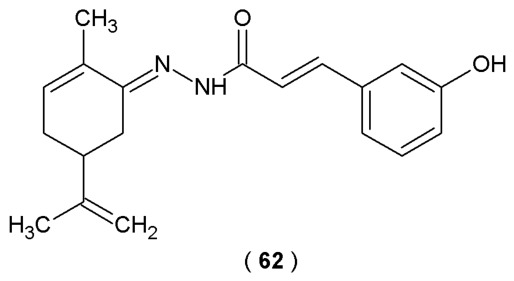
Acylhydrazone **62** with anti-inflammatory activity.

Compound **61** (part of the β-amino-*N*-acylhydrazones racemic mixture) was selected for further analysis due to its comparable potency to the *R*-enantiomer and its scalable synthesis. To ensure the reliability of the biological screening, the DPP-4 assay kit was first validated using (*R*)-sitagliptin as a standard. This molecule was selected as the most potent DPP-4 among the initial hybrid compounds due to its comparable potency to the *R*-enantiomer and its scalable synthesis. In a murine model of T2DM, compound **61** demonstrated antihyperglycemic effects and improved cardiac and renal dysfunction associated with metabolic syndromes (Table 24). The ability of β-amino-*N*-acylhydrazones molecules to prepare them as racemates or pure enantiomers provides a critical advantage for future medicinal chemistry efforts [59].

de Souza et al. describe the synthesis process of the terpene-cinnamoyl-acylhydrazone analogs. Scientists tested their effectiveness as pain-relieving and anti-inflammatory agents using animal models. Through molecular docking and computer simulations, the study explored how these molecules interact with CB1 and CB2 receptors and the TRPV1 channel. Based on the molecular structure of cannabidiol (CBD), novel derivatives utilize a specific acylhydrazone spacer to link a monoterpene moiety with a functionalized aromatic subunit. A series of twenty-six new terpene-cinnamoyl acylhydrazone analogs was derived from commercially available functionalized cinnamic acids. Scientists, to further elucidate the molecular mechanisms underlying the observed antinociceptive effects, conducted a molecular docking study on acylhydrazone **62**. Computational analysis aimed to characterize the potential interactions between compound **62** and the CB1, CB2, and TRPV1 receptors which are important factors in pain modulation and cannabinoid signaling. Experimental results highlighted compound **62** for its enhanced efficacy in alleviating neurogenic pain, outperforming the standard drug morphine (Figure 34). Biological evaluation confirmed a noticeable reduction in licking responses induced by formalin in mice treated with a single dose of 10 µmol/kg. Moreover, compound **62** almost completely suppresses the nociceptive response, demonstrating better anti-inflammatory activity than the control groups (morphine and acetylsalicylic acid). In summary, the novel terpene-cinnamoyl-*N*-acylhydrazone represents a promising candidate for the treatment of chronic pain without the typical central nervous system side effects [60].

According to the scientific literature findings acylhydrazones obtained on the basis of condensation reactions of hydrazides and aldehydes or ketones generally have lower toxicity than substrates for these reactions themselves. This is possibly due to the blockage effects of NH_2_ groups and CHO groups in the hydrazone moiety in comparison with free NH_2_ and CHO groups present in corresponding hydrazides as well as aldehydes, respectively [10,61,62,63,64,65,66,67].

## 3. Conclusions

In conclusion, certain limitations arising from the nature of the present work should be acknowledged. First and foremost, the limited chemical stability of the investigated compounds is notable, while another significant challenge is the need to ensure high selectivity toward therapeutic targets. Furthermore, future studies on acylhydrazones as potential neuroprotective drugs should consider pharmacokinetics, bioavailability, and the ability to cross the blood–brain barrier, as well as thoroughly assess toxicity and safety profiles, including possible long-term effects. However, the scope of these issues goes beyond the focus of the present review, which primarily concentrates on evaluating the effects of the investigated compounds at the *in vitro* level. The results of these basic studies provide a solid foundation for further research on structural optimization and safety, which are necessary for a comprehensive assessment of the translational potential of acylhydrazones in the treatment of neurodegenerative diseases.

In the review provided, all the studies were divided into three subgroups based on their biological effects of the acylhydrazones: cholinesterase inhibition, monoaminoxidase inhibition and other activities.

Notably, all research outcomes presented acylhydrazones as favorable chemical structures with multiple biological properties and as prospective agents for the treatment of various disorders, both neurological and beyond. Their structural versatility and target specificity as chemical compounds are proof of their suitability for tailored therapeutic strategies. All of the research results mentioned in this paper emphasize the role of acylhydrazones as a solid foundation for future structure–activity relationship (SAR) studies aimed at enhancing potency, selectivity, and safety. Given their chemical profile, they hold significant potential for molecular optimization in the neuroprotective therapeutics field, offering a strategic starting point for advanced medicinal chemistry efforts. Further optimization of multi-target directed ligands based on acylhydrazone moiety (MTDLs) could possibly lead to next-generation neuroprotective therapies for Parkinson’s and Alzheimer’s diseases and many other neurological diseases.

## Figures and Tables

**Table 1:** Inhibitory effect of acylhydrazones (**1**–**3**).

Compound	R	AChE % Inhibition (at 30 μM)	IC_50_ ± SD (μM)	BuChE % Inhibition (at 30 μM)	IC_50_ ± SD (μM)	% Inhibition of DPPH Radical (at 80 μM)	% Cell Viability (at 80 μM)
**1**	R^1^ = OH R^2^ = OCH_3_	47.1	13.04 ± 0.41	29.3	-	53.37	99.88
**2**	R^1^ = OH R^2^ = OH	43.0	-	62.0	21.99 ± 1.58	45.05	63.85
**3**	R^1^ = OCH_3_ R^2^ = OCH_3_	75.6	9.10 ± 0.68	3.4	-	1.41	80.70

IC_50_—Half maximal inhibitory concentration.

**Table 2:** Results of inhibitory effects of acylhydrazone (**4**) on AChE and BuChE.

Compound	AChE		BuChE		
	% Activity Inhibition	IC_50_ (µM)	% Activity Inhibition		IC_50_ (µM)
	100 µM		10 µM	100 µM	
**4**	24.95 ± 1.37	>100	73.02 ± 1.17	77.81 ± 0.87	0.07 ± 0.02
Donepezil	-	0.01 ± 0.003	-	-	3.54 ± 0.54

**Table 3:** Data of inhibitory effects of acylhydrazone **5**.

Compound	DPPH Scavenger IC_50_ (µmol/L)	MPO Inhibition IC_50_ (µmol/L)	AChE Inhibition (%, 100 µmol/L)
**5**	42.4 ± 1.9	5.3 ± 0.5	54.2 ± 1.7
DMSO	-	-	7.0 ± 7.1
Isoniazide	22.9 ± 0.7	3.9 ± 0.3	5.8 ± 12.5
Quercetin	15.3 ± 0.6	-	-

**Table 4:** Potential affinity of acylhydrazone **6** confirmed by computational pharmacodynamic profile.

Compound 6	
BBB permeability [log BBB]	0.07
Microtubule-associated protein tau substrate similarity [%]	20.0
COX-1/COX-2 substrate similarity [%]	40.0

**Table 5:** AChE and BuChE activity of acylhydrazone (**7**) and data of its potential antioxidant activity.

Compounds	IC_50_ ± SEM (µM)			Reduction in DPPH (%) Inhibition at 100 µM IC_50_ ± SEM (µM)
	AChE	BuChE	Selectivity (SI)	
**7**	1.00 ± 0.43	2.04 ± 0.69	2.03	14.01 ± 1.33 (>100)
Donepezil	0.04 ± 0.01	1.87 ± 0.08	81.3	5.4 ± 1.11 (>500)
Tacrine	0.05 ± 0.01	0.007 ± 0.00	0.14	17.2 ± 1.19 (>500)
Ascorbic acid	-	-	-	98.26 ± 1.88 (9.01 ± 1.33)

**Table 6:** The inhibitory effect data of acylhydrazone **8** on SOICR and AChE.

Compound	SOICR Inhibition (%)			AChE Inhibition (%)
	0.1 μM	3 μM	10 μM	5 μM
**8**	24.1 ± 0.2	50.3 ± 0.3	73.6 ± 0.2	29.1 ± 0.3
Dantrolene	30.8 ± 0.1	73.1 ± 0.1	94.2 ± 0.2	-
Donepezil	-	-	-	99.5 ± 0.1

**Table 7:** The IC_50_ values of acylhydrazone **9** and the initial compound **SM3**.

Compound	AChE IC_50_ (µM) ± SD	BuChE IC_50_ (µM) ± SD
**SM3**	23.1 ± 0.6540	21.8 ± 0.8761
**9**	27.8 ± 0.7232	19.0 ± 0.8625
Galantamine	29.5 ± 0.9036	27.8 ± 0.8740

**Table 8:** The IC_50_ values for bis(acylhydrazones) **10**, **11**, **12** and **13**.

Compound	AChE IC_50_ (µM)	BuChE IC_50_ (µM)
**10**	25.6 ± 1.4	22.0 ± 1.1
**11**	26.3 ± 0.4	276.7 ± 0.6
**12**	28.4 ± 0.5	281.6 ± 0.8
**13**	45.2 ± 1.0	31.3 ± 1.3
Galantamine	29.5 ± 0.9	27.8 ± 0.8

**Table 9:** The results of the inhibitory activity study of acylhydrazone (**15**).

Compound	AChE (IC_50_, μM)	BuChE (IC_50_, μM)	DPPH (IC_50_, μM)	MAO-A (IC_50_, μM)	MAO-B (IC_50_, μM)
**15**	0.00098 ± 0.000001	35.84 ± 4.5	991.45 ± 11.40	203.91 ±17.9	346.03 ± 14.3
Donepezil	0.12297 ± 0.0.0103	-	-	-	-
Rivastigmine	-	5.88 ± 0.64	-	-	-
Quercetin	-	-	491.23 ± 14.8	-	-
Biperiden	-	-	-	-	237.59 ± 16.3
Methylene blue	-	-	-	143.6 ± 22.1	-

**Table 10:** Inhibitory activity data of acylhydrazones **16** and **17**.

Compound	AChE	BuChE	SI
	Ki (µM)	Ki (µM)	
**16**	89 ± 5	68 ± 3	1.3
**17**	102 ± 3	38 ± 6	2.7
Galantamine	0.52 ± 0.03	1.08 ± 0.08	0.48
Donepezil	0.024 ± 0.007	2.33 ± 0.73	0.010

K_i_—enzyme-inhibitor dissociation constants.

**Table 11:** Inhibitory activity data of acylhydrazone (**18**, **19**).

Compound	IC_50_ (µM)			
	hCA I	hCA II	AChE	BuChE
**18**	102.7 ± 2.05	97.4 ± 1.95	12.1 ± 0.24	83.6 ± 1.67
**19**	30.4 ± 0.61	23.2 ± 0.46	61.4 ± 1.23	76.4 ± 1.53
Acetazolamide	286.66 ± 2.42	26.63 ± 0.38	-	-
Neostigmine	-	-	135.90 ± 1.86	84.0 ± 1.07
Rivastigmine	-	-	60.00 ± 0.73	14.10 ± 0.35

**Table 12:** The results of the inhibitory activity study for compound **20**.

Compound	AChE Inhibition IC_50_ (μM)	BACE-1 Inhibition IC_50_ (μM)	Aβ Fibryl Formation Inhibition IC_50_ (μM)	Radical Scaveging Activity (%)
**20**	2.98	63.9	17.1	88
Tacrine	0.3	-	-	-
Curcumin	-	-	6.9	-
Quercetin	-	5.6	-	-
Ascorbic acid	-	-	-	88

**Table 13:** The *in vitro* activity against acetyl- and butyrylcholinesterase for acylhydrazones (**21**–**27**).

Compound	R	AChE IC_50_ (µM)	BuChE IC_50_ (µM)	Selectivity
**21**	4-hydroxyphenyl	105.9 ± 0.2	53.9 ± 2.6	BuChE inhibitor
**22**	3,4,5-trimethoxyphenyl	66.3 ± 1.3	70.6 ± 1.4	AChE inhibitor
**23**	3-methoxy-4-hydroxyphenyl	115.7 ± 1.2	59.3 ± 0.3	BuChE inhibitor
**24**	2-methoxy-3-hydroxyphenyl	62.3 ± 0.6	90.1 ± 0.7	AChE inhibitor
**25**	3,4-dimethoxyphenyl	67.3 ± 1.3	51.0 ± 1.2	Dual inhibitor
**26**	2-hydroxyphenyl	69.5 ± 0.9	57.1 ± 0.8	Dual inhibitor
**27**	3-ethoxy-4-(3-(2-ethoxy-4-formylphe-noxy)propoxy)phenyl	70.5 ± 1.3	53.2 ± 1.6	Dual inhibitor
Galantamine	standard inhibitor	69.5 ± 0.9	57.1 ± 0.8	AChE inhibitor

**Table 14:** The IC_50_ values for compounds **ZINC4372573, 28** and the reference substance.

Compound	AChE IC_50_ (μM)	BuChE IC_50_ (μM)	SI
**28**	0.18 ± 0.06	7.61 ± 0.21	95.13
**ZINC4372573**	15.53 ± 0.12	57.15 ± 0.72	3.68
Galantamine	3.65 ± 0.01	15.29 ± 0.04	4.19

**Table 15:** The results of the *in vitro* study of acylhydrazones **29** and **30**.

Compound	Inhibition Activity at 10 μM (%)		IC_50_ (μM)		SI
	MAO-A	MAO-B	MAO-A	MAO-B	
**29**	61.76 ± 6.24	11.74 ± 1.42	23.42 ± 0.56	0.14 ± 0.011	167.29
**30**	63.75 ± 1.76	14.41 ± 1.20	19.57 ± 1.41	0.15 ± 0.02	130.47
Toloxatone			1.646 ± 0.094		
Lazabemide				0.073 ± 0.0013	
Clorgyline			0.0079 ± 0.00094		
Pargyline				0.11 ± 0.011	

**Table 16:** Monoamine oxidase inhibition activity data for compounds (**37**–**39**).

Compound	Inhibition Activity at 10 µM (%)		IC_50_ (µM)		SI
	MAO-A	MAO-B	MAO-A	MAO-B	
**37**	62.04 ± 1.87	8.53 ± 1.71	32.711 ± 0.210	0.124 ± 0.015	263.80
**38**	50.76 ± 7.29	3.20 ± 0.77	19.176 ± 5.960	0.082 ± 0.010	233.85
**39**	62.28 ± 0.30	5.66 ± 1.75	22.107 ± 0.063	0.104 ± 0.005	212.57
Toloxatone			1.080 ± 0.025	-	
Lazabemide			-	0.110 ± 0.016	
Clorgyline			0.007 ± 0.0007	-	
Pargyline			-	0.140 ± 0.0059	

**Table 17:** The results of inhibitory activity assays of acylhydrazones (**40**–**42**) and reference substances.

Compound	MAO-A % Inhibition (10^−3^/10^−4^ M)	MAO-A IC_50_ (µM)	MAO-B % Inhibition (10^−3^/10^−4^ M)	MAO-B IC_50_ (µM)
**40**	42.085 ± 0.985/ 28.156 ± 0.702	>1000	76.759 ± 1.154/ 42.950 ± 0.851	>100
**41**	41.465 ± 0.986/ 20.057 ± 0.722	>1000	74.036 ± 1.570/ 45.145 ± 0.914	>100
**42**	41.347 ± 0.708/ 24.079 ± 0.958	>1000	69.182 ± 1.046/ 39.357 ± 0.733	>100
Moclobemide	94.121 ± 2.760/ 82.143 ± 2.691	6.061 ± 0.262	-	-
Selegiline	-	-	98.258 ± 1.052/ 96.107 ± 1.165	0.037 ± 0.001

**Table 18:** The IC_50_ and selectivity index values for acylhydrazones (**43**–**46**).

Compound	MAO-A IC_50_ (μM) ± SD	MAO-B IC_50_ (μM) ± SD	SI
**43**	0.598 ± 0.09	0.662 ± 0.10	0.903
**44**	0.978 ± 0.20	0.524 ± 0.20	1.866
**45**	0.488 ± 0.10	0.633 ± 0.10	0.771
**46**	0.561 ± 0.20	0.611 ± 0.20	0.918
Selegiline	-	0.320 ± 0.09	
Chlorgyline	0.355 ± 0.09	-	

**Table 19:** The IC_50_ values for compound **47,** its hydrochloride and the reference substance.

Compound	Mitogen-Activated Protein Kinase—p38 MAPK IC_50_ (μM)
**47**	40.6
**47 hydrochloride**	28.4
SB 203580 (inhibitor of p38 MAPK)	0.075

**Table 20:** The inhibitory activity data for potential ATX inhibitors—acylhydrazones (**51**–**53**).

Compound	Type/Class	ATX IC_50_ (nM)	Type of Assay
**51**	Dual Inhibitor	38.4	Enzymatic Assay
**52**	Dual Inhibitor	29.1	Enzymatic Assay
**53**	Dual Inhibitor	24.2	Enzymatic Assay
PF-8380	Type I Inhibitor	3	LPC Assay
GLPG1690 (Ziritaxestat)	Type IV Inhibitor	131	LPC Assay

**Table 21:** Activity data of acylhydrazone **56**.

Compound	Activity	Selectivity
*N*-Alkyl isatin acylhydrazone **56**	K_i_ CB2R = 44.3 ± 10.2 nM EC_50_ CB1R = 867.0 ± 1.1 nM EC_50_ CB2R = 63.4 ± 1.3 nM	CB2 agonist

K_i_—enzyme-inhibitor dissociation constants; EC_50_—half maximal effective concentration.

**Table 22:** The IC_50_ values for compound **57** and acarbose.

Compound	IC_50_ (µM)
**57**	1.08 ± 0.34
Acarbose	575.76 ± 12.31

**Table 23:** Acylhydrazones **58**, **59** and **60** with anti-inflammatory properties.

Compound	IC_50_ (μM)				SI
	COX-1	COX-2	15-LOX	mPGES-1	
**58**	12.23 ± 0.1	0.049 ± 0.0	1.72 ± 0.03	4.10 ± 0.1	250
**59**	9.82 ± 0.1	0.089 ± 0.0	3.05 ± 0.1	4.90 ± 0.2	110
**60**	11.12 ± 0.1	0.057 ± 0.01	2.39 ± 0.03	2.80 ± 0.1	195
Celecoxib	14.7 ± 0.2	0.045 ± 0.0	-	20.10 ± 0.1	327
Indomethacin	0.1 ± 0.01	0.080 ± 0.0	-	-	1.25
Diclofenac sodium	3.8 ± 0.03	0.84 ± 0.01	-	-	4.52
Quercetin	-	-	3.34 ± 0.1	-	-

**Table 24:** Inhibitory effect on DPP-4 by acylhydrazone **61** and sitagliptin.

Compound	Inhibition of DPP-4 IC_50_ (μM)
**61**	10.6
Sitagliptin	0.092

## Data Availability

No new data were created or analyzed in this study. Data sharing is not applicable to this article.

## Abbreviations

15-LOX	Lipoxygenase
3-NP	Neurotoxin 6-hydroxydopamine
6-OHDA	6-hydroxydopamine hydrochloride
Aβ	β-amyloid
ACh	Acetylcholine
AChE	Acetylcholinesterase
AD	Alzheimer’s disease
ALS	amyotrophic lateral sclerosis
Amplex UltraRed reagent	10-acetyl-3,7-dihydroxy-phenoxazine
APP	amyloid precursor protein
ATX	Autotaxin
BACE1	precursor protein cleaving enzyme 1
BBB	blood–brain barrier
BHT	Butylated hydroxytoluene
BSA	Bovine serum albumin
BuChE	Butyrylcholinesterase
CNS	Central nervous system
COMT	catechol-*O*-methyltransferase
COX-1	Cyclooxygenase-1
COX-2	Cyclooxygenase-2
CREB	cAMP response element-binding protein
DA	dopamine
DAT	Dopamine transporter
DCFH-DA	2′,7′-dichlorodihydrofluorescein diacetate
DCL	Dynamic combinatorial library
DPP-4	Dipeptidyl peptidase IV inhibitors
DPPH	2,2-Diphenyl-1-picrylhydrazyl—the radical scavenging assay
DMSO	dimethylsulfoxide
EC_50_	Half maximal effective concentration
EIS	electrochemical impedance spectroscopy
ERRγ	Estrogen-Related Receptor Gamma (γ)
FAAH	Human Fatty Acid Amide Hydrolase
FAD	Flavin adenine dinucleotide
FRAP	Ferric reducing antioxidant power
Glu	amino acid glutamate
GPCR	G protein-coupled receptor
GSH	Glutathione
HD	Huntington’s disease
hCA I	Carbonic Anhydrase I
hCA II	Carbonic Anhydrase II
hMAO-B	recombinant human MAO-B
HTS	high-throughput screening assay
IAChE	Cholinesterase inhibitors
IC_50_	Half Maximal Inhibitory Concentration
IFN-γ	Interferon gamma (γ)
IL-1	Interleukin-1
IL-10	Interleukin-10
IL-12	Interleukin-12
IL-17	Interleukin-17
IL-6	Interleukin-6
LPA	Lysophosphatidic acid
LPC	Lysophosphatidylcholine
MAPK	p38 Mitogen-Activated Protein Kinase
MD	Molecular dynamics
MEK-ERK	Mitogen-activated protein kinase cascade
mPGES	Microsomal prostaglandin E2 synthase 1
MPO	Myeloperoxidase
NBT	nitroblue tetrazolium
Neuro-2a cells	Neuroblastoma cell line
NO	Nitric oxide
NSPCs	Neuronal stem and progenitor cells
OAβ1–42	Soluble oligomers with neurotoxic effects
PAMPA	Parallel Artificial Membrane Permeability Assay
PAS	Peripheral Anionic Site
PD	Parkinson’s disease
PDE	phosphodiesterase
PI3K-Akt	Phosphoinositide-3-kinase — protein kinase B/Akt pathway
RAW 264.7 cells	Murine macrophage cell line
ROS	Reactive Oxygen Species
SH-SY5Y	Neuroblastoma cancer cell lines
SI	Selectivity index
SOICR	Store Overload-induced Ca^2+^ Release
SV	Synaptosomal viability
*t*-BuOOH	*tert*-butyl hydroperoxide
TH	Tyrosine hydroxylase
TNF-α	Tumor Necrosis Factor Alpha
